# Fasudil hydrochloride and ozagrel sodium combination therapy for patients with aneurysmal subarachnoid hemorrhage: a cross-sectional study using a nationwide inpatient database

**DOI:** 10.1186/s40780-024-00370-w

**Published:** 2024-08-13

**Authors:** Hiroshi Magara, Takuaki Tani, Shinobu Imai, Anna Kiyomi, Kiyohide Fushimi, Munetoshi Sugiura

**Affiliations:** 1 Department of Drug Safety and Risk Management, School of Pharmacy, Tokyo University of Pharmacy and Life Sciences, 1432-1, Horinouchi, Hachioji, Tokyo, 192-0392 Japan; 2 Department of Pharmacoepidemiology, Showa University Graduate School of Pharmacy, Tokyo, Japan; 3 Department of Health Policy and Informatics, Tokyo Medical and Dental University Graduate School of Medicine, Tokyo, Japan

**Keywords:** Subarachnoid hemorrhage, Fasudil hydrochloride, Ozagrel sodium, Cross-sectional study, In-hospital mortality

## Abstract

**Background:**

Fasudil and ozagrel are drugs with the same indications for the treatment of cerebral vasospasm in Japan. However, there have been no definitive conclusions on the clinical efficacy of fasudil hydrochloride and ozagrel sodium monotherapy or their combination. Therefore, we aimed to investigate the effectiveness of the combined administration of fasudil hydrochloride and ozagrel sodium in Japanese patients with subarachnoid hemorrhage (SAH).

**Methods:**

This cross-sectional study used Diagnosis Procedure Combination data to assess patients who were hospitalized with SAH and received fasudil hydrochloride or ozagrel sodium between April 2016 and March 2020 (*n* = 17,346). The participants were divided into three groups based on the treatment received: fasudil hydrochloride monotherapy (F group, *n* = 10,484), ozagrel sodium monotherapy (O group, *n* = 465), and fasudil hydrochloride and ozagrel sodium combination therapy (FO group, *n* = 6,397). The primary outcome was in-hospital mortality. Multivariable adjusted logistic regression analysis (significance level, 5%) was used for data analyses.

**Results:**

The results of the multivariable analysis, adjusted for factors considered to impact prognosis, showed that the adjusted odds ratio (OR) with the F group as the reference for in-hospital mortality was 0.94 in the FO group (95% confidence interval [CI]: 0.81–1.08, *p* = 0.355), with no differences compared to the F group.

**Conclusion:**

Fasudil hydrochloride and ozagrel sodium had different mechanisms of action, suggesting a synergistic effect of combination therapy. However, a comparison of fasudil hydrochloride monotherapy and combination therapy of fasudil hydrochloride and ozagrel sodium showed no difference in the prognostic effect. Therefore, it was suggested that fasudil hydrochloride monotherapy may be sufficient.

**Supplementary Information:**

The online version contains supplementary material available at 10.1186/s40780-024-00370-w.

## Background

Subarachnoid hemorrhage (SAH) can be categorized as either traumatic or non-traumatic. Aneurysmal SAH (aSAH) is the most common form of non-traumatic SAH, accounting for 85% of all reported cases of SAH [1]. The incidence of aSAH is reported to be 1.6-fold higher in women than in men and generally peaks between 50 and 60 years of age, although it can also occur in younger individuals [1]. Cerebral vasospasm has been reported to occur at a frequency of 30–70%, mainly 4–14 days after the onset of aSAH [2]. In addition, no improvement in in-hospital mortality after aSAH onset has been observed globally (19–20% in 2021) and in the USA (13.7% in 2006 to 13.1% in 2018) [3]. In Japan, the rate of favorable outcomes after SAH did not change from 2000 to 2019 [4]. Therefore, improved treatment options after the onset of aSAH are required.

The calcium channel blocker nimodipine is approved by the European Medicines Agency and USA Food and Drug Administration, and its use is recommended for the prevention of delayed ischemic deficit by the European Stroke Organization Guidelines for Management of Intracranial Aneurysms and Subarachnoid Hemorrhage [5] and 2023 American Heart Association/American Stroke Association Guidelines for the Management of Patients with Aneurysmal Subarachnoid Hemorrhage [3]. However, nimodipine is not yet approved for use in Japan.

Fasudil hydrochloride (a rho kinase inhibitor) [6] and ozagrel sodium (a thromboxane synthase inhibitor) [7] have been approved in Japan as therapeutic agents for cerebral vasospasm after SAH. According to the Stroke Treatment Guideline 2021, Japan, the administration of fasudil hydrochloride and ozagrel sodium as systemic pharmacotherapy is a moderate recommendation (Grade B, low level of evidence), and there are no strongly recommended treatments [8]. However, although it has the same indication, reports on fasudil hydrochloride and ozagrel sodium combination therapy are scarce. In mice, the combination of fasudil hydrochloride and ozagrel sodium has been reported to have an inhibitory effect on cerebral infarction compared with single-agent administration [9]. In the post-marketing surveillance of fasudil hydrochloride, the combination of fasudil hydrochloride and ozagrel sodium was well tolerated but did not result in better efficacy than fasudil hydrochloride monotherapy [10]. In addition, the combination therapy of fasudil hydrochloride and ozagrel sodium has superior effectiveness over ozagrel monotherapy [11]. Evidence on the efficacy of both drugs as a monotherapy or in combination is inconclusive.

Therefore, we conducted this cross-sectional study using Diagnosis Procedure Combination (DPC) data to investigate the effectiveness of the combined administration of fasudil hydrochloride and ozagrel sodium in Japanese patients with SAH.

## Methods

### Data source

The nationwide DPC database collects administrative claims and abstract data on discharges from more than 1200 acute care hospitals in Japan [12, 13]. The data obtained include the unique hospital identifier, patient administrative claims data, and information regarding age, sex, diagnoses at admission (coded according to the International Classification of Diseases, 10th Revision [ICD-10]), medical history, medication (see Additional file 1), treatment procedure, length of stay, Japan Coma Scale (JCS) score at admission [14] (see Additional file 2), modified Rankin Scale (mRS) score at discharge, and complications.

### Study population

This was a cross-sectional study of DPC data from April 2016 to March 2020. We selected patients admitted with SAH due to ruptured aneurysms (using the ICD-10 code associated with SAH: I60.0-9; see Additional file 3), because there are validation reports in acute hemorrhagic stroke patients using this code [15]. Patients who underwent clipping or coiling treatment for SAH were aged > 18 years and were hospitalized for up to 7 days after the onset of SAH were included. Patients who died within the first 24 h of admission, those with unknown mRS scores before stroke and at discharge, and those who did not receive fasudil hydrochloride or ozagrel sodium were excluded. The included patients were divided into three groups based on the treatment received: fasudil hydrochloride monotherapy, ozagrel sodium monotherapy, and fasudil hydrochloride and ozagrel sodium combination therapy (F, O, and FO groups, respectively). Combination therapy of fasudil hydrochloride and ozagrel sodium was defined as an overlap (not switching) between the days of administration of both drugs for more than 1 day. According to the package insert, the typical dosing schedules for fasudil hydrochloride and ozagrel sodium are as follows: 30 mg of fasudil hydrochloride is administered intravenously 2–3 times a day for 14 days, and 80 mg per day of ozagrel sodium is continuously administered intravenously for 14 days.

### Outcome measures

The primary outcome was in-hospital mortality, and the secondary outcome was the mRS score at discharge. Functional independence was defined as mRS score ≤ 2. When comparing different treatment options for cerebral vasospasm and their effect on poor outcomes (mRS 3–6 at discharge), fasudil hydrochloride was associated with lower proportion of in-hospital mortality and poor outcomes at discharge [16]. Moreover, as cerebral vasospasms have been reported to occur within approximately 2 weeks of the onset of aSAH [2], we considered that the outcome at discharge was sufficient to verify the therapeutic efficacy of the drug.

Patient age, sex, comorbidities, Charlson Comorbidity Index (CCI), JCS score at admission, Glasgow Coma Scale (GCS) score at admission, mRS score at discharge, treatment method, aneurysm location, days from the onset of SAH to admission, presence of ambulance transport, presence of intensive care unit use, use of mechanical ventilation, and concomitant medications were analyzed. The GCS score was calculated by converting from the JCS score [17] (see Additional file 4). Age was categorized as either < 75 years or ≥ 75 years because previous studies have shown that the clinical outcomes of patients after aSAH greatly differed when the cut-off age was set at 75 years [18]. Concomitant medications were aggregated for representative medications administered to patients with subarachnoid hemorrhage who were administered at the institution during their hospital stay. Diabetes mellitus (ICD-10 code: E10-14), hypertension (I10-15), dyslipidemia (E785), cerebral infarct (I63), and cerebral hemorrhage (I61) were evaluated as complications at admission (see Additional file 3). Cerebral infarction (I63) and cerebral hemorrhage (I61) were evaluated as events that occurred during hospitalization (see Additional file 3). For these ICD-10 codes, the accuracy was reported as it was used when scoring comprehensive stroke care capabilities [19]. Quan’s algorithm was used for calculating the CCI [20]. Days from onset of SAH to admission were categorized as either ≤ 3 days or 4–7 days. The location of the ruptured cerebral aneurysms was classified as either the anterior communicating artery, middle cerebral artery, internal carotid artery, anterior cerebral artery, vertebral artery, basilar artery, or others based on the ICD-10 codes. Hospital case volume was classified into quartiles (Q1–Q4) based on the case volume per 4 years of clipping or coiling for SAH.

### Statistical analysis

For the comparison of patient backgrounds, the chi-square test was used for categorical variables and the one-way analysis of variance for continuous variables. Statistical analysis was performed using multivariable-adjusted logistic regression analysis (significance level, 5%). Additionally, subgroup analysis was performed as sensitivity analysis and for the assessment of selection bias for < 75-year and ≥ 75-year subgroups, as well as coiling and clipping treatment subgroups. The following variables were evaluated as multivariable factors potentially affecting prognosis: age, sex, treatment procedure for SAH (clipping, coiling), starting time of treatment (Day 1, Day 2, Day 3 and ≥ Day 4), JCS score on admission (0, 1-digit code, 2-digit code, 3-digit code), mRS score before onset of stroke, days from onset of SAH to admission (≤ 3 days, 4–7 days), ambulance use, CCI (0, ≥ 1), presence of comorbidities (hypertension, diabetes mellitus, dyslipidemia, cerebral infarct, cerebral hemorrhage), concomitant medications (cilostazol, statins, edaravone, antihypertensive drug, antiplatelet drug), use of a specific intensive care unit, use of mechanical ventilation, and hospital case volume (≤ 7, 8–17, 18–33, and ≥ 34 cases/4 years). Patient-specific information was submitted as DPC data after anonymization at individual medical institutions. To examine the impact of patients with multiple admissions, patient ID information for multiple admissions in the same hospital during the period was included as a factor for analysis. All statistical analyses were performed using the RStudio (version 4.2) software (R Foundation for Statistical Computing, Vienna, Austria, http://www.R-project.org). This study followed the Strengthening the Reporting of Observational Studies in Epidemiology (STROBE) guidelines.

## Results

**Fig. 1:**
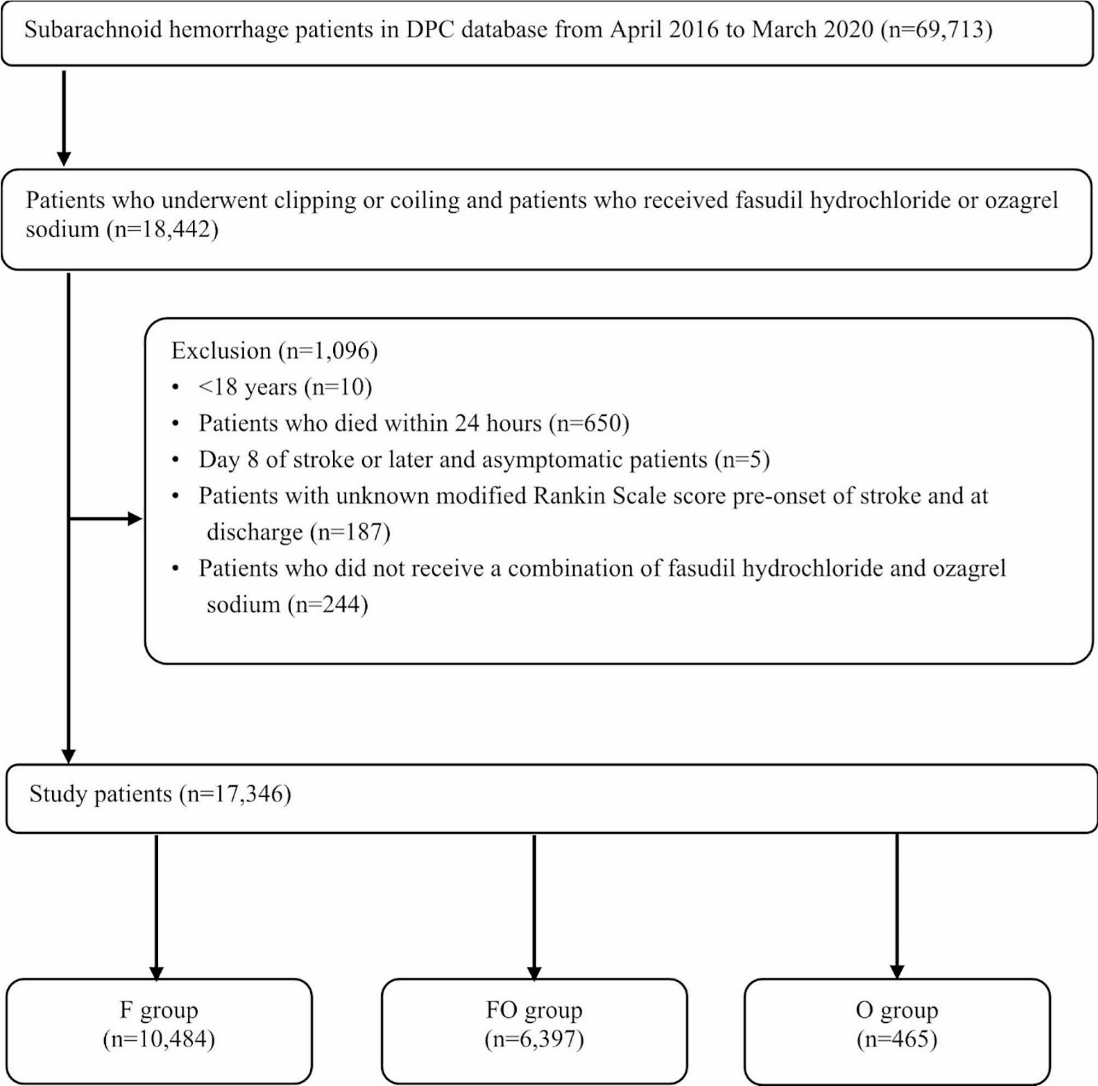
Study flowchart. DPC, Diagnosis Procedure Combination; F group, fasudil hydrochloride monotherapy; FO group, fasudil hydrochloride and ozagrel sodium combination therapy; O group, ozagrel sodium monotherapy

We identified 17,346 individuals with SAH (F group, *n* = 10,484; FO group, *n* = 6,397; O group, *n* = 465) at 756 hospitals based on the relevant ICD-10 code (Fig. 1). The starting time and duration of treatment of fasudil hydrochloride and ozagrel sodium are shown in Table 1. These drugs were started on Day 3–5 after onset of SAH, the frequent period for cerebral vasospasm, and the treatment duration was 11.3 ± 3.4 days in the F group, 10.1 ± 3.7 days in the FO group, and 7.7 ± 6.7 days in the O group.

**Table 1:** Starting time and treatment duration of fasudil hydrochloride and ozagrel sodium

		F group	FO group	O group
Starting time of treatment (day)	Mean ± SD	3.5 ± 2.5	4.2 ± 2.5	4.8 ± 5.9
	Median	3.0	3.0	3.0
Treatment duration (day)	Mean ± SD	11.3 ± 3.4	10.1 ± 3.7	7.7 ± 6.7
	Median	12.0	11.0	9.0

Table 2 summarizes the demographic characteristics of patients in each group (see Additional file 5, including the non-administration of fasudil hydrochloride and ozagrel sodium). The mean age was 63.6 (standard deviation [SD]: 14.4), 63.0 (SD: 14.4), and 64.4 (SD: 14.8) years in the F, FO and O groups. The O group had fewer patients who underwent clipping and a higher ratio of those who underwent coiling than the F and FO groups (clipping: F group 60.5%, FO group 57.5%, O group 46.0%; coiling: F group 38.1%, FO group 40.8%, O group 53.1%, *p* < 0.001). The mRS scores from before admission did not differ between the groups. Regarding hospital case volume, the only notable difference was that the O group tended to have fewer patients from high-volume centers. Regarding concomitant medication use, fewer patients in the O group concomitantly used cilostazol (F group, 48.1%; FO group, 50.2%; O group, 23.2%, *p* < 0.001) and statins (F group, 37.9%; FO group, 40.0%; O group, 18.3%, *p* < 0.001), and more used edaravone (F group, 28.2%; FO group, 34.5%; O group, 41.3%, *p* < 0.001), than in the other two groups.

**Table 2:** Baseline characteristics of patients in the F, FO, and O groups

	F group (*n* = 10,484)	FO group (*n* = 6,397)	O group (*n* = 465)	*p*-value
Age, years, mean (SD)	63.6 (14.4)	63.0 (14.4)	64.4 (14.8)	0.005
Age group				0.020
< 75 years, n (%)	7779 (74.2)	4854 (75.9)	335 (72.0)	
≥ 75 years, n (%)	2705 (25.8)	1543 (24.1)	130 (28.0)	
Sex, n (%)				0.143
Male	3157 (30.1)	1938 (30.3)	160 (34.4)	
Female	7327 (69.9)	4459 (69.7)	305 (65.6)	
Location of aneurysms, n (%)				
ICA	2921 (27.9)	1856 (29.0)	132 (28.4)	0.272
MCA	2456 (23.4)	1412 (22.1)	83 (17.8)	0.005
AcomA	2528 (24.1)	1646 (25.7)	113 (24.3)	0.060
PcomA	160 (1.5)	109 (1.7)	13 (2.8)	0.087
BA	397 (3.8)	250 (3.9)	30 (6.5)	0.015
VA	713 (6.8)	418 (6.5)	43 (9.2)	0.078
Other	1578 (15.1)	854 (13.4)	65 (14.0)	0.009
Treatment modality, n (%)				< 0.001
Clipping	6347 (60.5)	3679 (57.5)	214 (46.0)	
Coiling	3997 (38.1)	2611 (40.8)	247 (53.1)	
Clipping and coiling	140 (1.3)	107 (1.7)	4 (0.9)	
Ambulance use, n (%)	9069 (86.5)	5593 (87.4)	383 (82.4)	0.005
Days from onset of SAH to admission, n (%)				< 0.001
≤ 3 days	10,155 (96.9)	6248 (97.7)	442 (95.1)	
4–7 days	329 (3.1)	149 (2.3)	23 (4.9)	
ICU admission, n (%)	4842 (46.2)	2921 (45.7)	216 (46.5)	0.788
Artificial ventilation, n (%)	6359 (60.7)	3928 (61.4)	243 (52.3)	< 0.001
Length of hospital stay (SD)	48.1 (46.9)	48.7 (40.0)	45.4 (39.3)	0.256
Hospital case volume quartiles, cases/4 years, n (%)				< 0.001
1–7	428 (4.1)	247 (3.9)	35 (7.5)	
8–17	1278 (12.2)	972 (15.2)	119 (25.6)	
18–33	2938 (29.0)	1516 (23.7)	119 (25.6)	
≥ 34	5840 (55.7)	3662 (57.2)	192 (41.3)	
JCS score at admission, n (%)				0.248
0	2041(19.5)	1271 (19.9)	104 (22.4)	
1-digit code	2897 (27.6)	1768 (27.6)	129 (27.7)	
2-digit code	2496 (23.8)	1593 (24.9)	106 (22.8)	
3-digit code	3050 (29.1)	1765 (27.6)	126 (27.1)	
GCS, n (%)				0.053
15	3788 (36.1)	2308 (36.1)	187 (40.2)	
14	614 (5.9)	363 (5.7)	23 (4.9)	
13	536 (5.1)	368 (5.8)	23 (4.9)	
12 − 7	3266 (31.2)	2080 (32.5)	135 (29.0)	
6 − 3	2280 (21.7)	1278 (20.0)	97 (20.9)	
mRS score before onset of stroke, n (%)				< 0.001
0	7810 (74.5)	4767 (74.5)	320 (68.8)	
1	1189 (11.3)	792 (12.4)	74 (15.9)	
2	431 (4.1)	267 (4.2)	32 (6.9)	
3	286 (2.7)	138 (2.2)	14 (3.0)	
4	310 (3.0)	167 (2.6)	6 (1.3)	
5	458 (4.4)	266 (4.2)	19 (4.1)	
Charlson Comorbidity Index, n (%)				< 0.001
0	6640 (63.3)	4247 (66.4)	310 (66.7)	
≥ 1	3844 (36.7)	2150 (33.6)	155 (33.3)	
Comorbidities, n (%)				
Hypertension	5995 (57.2)	3645 (57.0)	287 (61.7)	0.135
Diabetes	1027 (9.8)	607 (9.5)	45 (9.7)	0.807
Hyperlipidemia	1237 (11.8)	775 (12.1)	43 (9.2)	0.176
Cerebral infarction	617 (5.9)	471 (7.4)	38 (8.2)	< 0.001
Cerebral hemorrhage	341 (3.3)	170 (2.7)	15 (3.2)	0.089
Concomitant medication, n (%)				
Cilostazol	5041 (48.1)	3209 (50.2)	108 (23.2)	< 0.001
Statins	3976 (37.9)	2558 (40.0)	85 (18.3)	< 0.001
Edaravone	2961 (28.2)	2208 (34.5)	192 (41.3)	< 0.001
Catecholamine	778 (7.4)	537 (8.4)	51 (11.0)	0.003
Antihypertensive drug	9164 (87.4)	5631 (88.0)	347(74.6)	< 0.001
Antiplatelet drug	2627 (25.1)	1924 (30.1)	179 (38.5)	< 0.001

F group: fasudil hydrochloride, FO group: combination of fasudil hydrochloride and ozagrel sodium, O group: ozagrel sodium

AcomA: anterior communicating artery, BA: basilar artery, GCS: Glasgow Coma Scale, ICA: internal carotid artery, ICU: intensive care unit, JCS: Japan Coma Scale, MCA: middle cerebral artery, mRS: modified Rankin Scale, PcomA: posterior communicating artery, SD: standard deviation, VA: vertebral artery

Concomitant medications: All medications were administered during hospitalization

Antihypertensive drug: Antihypertensive agents (Aspirin, Dipyridamole, Ticlopidine, Clopidogrel and Prasugrel) used for acute treatment after subarachnoid hemorrhage

The proportion of in-hospital mortality was 6.0% in the F group, 5.7% in the FO group, and 12.9% in the O group (*p* < 0.001, Table 3) and, thus, it tended to be higher in the O group than in the F and FO groups. The proportion of patients with mRS score ≤ 2 at discharge was 52.4% in the F group, 51.9% in the FO group, and 49.5% in the O group (*p* = 0.403); although this was not significantly different between the groups, it tended to be lower in the O group (Table 3).

The results of the multivariable analysis adjusted for factors considered to have an impact on prognosis showed that the adjusted odds ratio (OR) with the F group as the reference for in-hospital mortality was 0.94 in the FO group (95% confidence interval [CI]: 0.81–1.08, *p* = 0.355), with no differences compared to the F group. That for the O group was 2.05 (95%CI: 1.50–2.80, *p* < 0.001) and tended to be higher in the O group compared to that in the F group (Table 4). The adjusted OR for the proportion of mRS score ≤ 2 at discharge was 0.93 in the FO group (95% CI: 0.86–1.00, *p* = 0.054). That for the O group was 0.95 (95% CI: 0.76–1.18, *p* = 0.625) and tended to be lower in the O group compared to that in the F group (Table 5).

**Table 3:** Crude outcomes in the F, FO, and O groups

	F group	FO group	O group	*p*-value
All, N	10,484	6,397	465	
In-hospital mortality, n (%)	626 (6.0)	366 (5.7)	60 (12.9)	<0.001
mRS score ≤ 2 at discharge, n (%)	5,496 (52.4)	3,320 (51.9)	230 (49.5)	0.403
Age < 75 years				
N	7,779	4,854	335	
In-hospital mortality, n (%)	378 (4.9)	226 (4.7)	41 (12.2)	< 0.001
mRS score ≤ 2 at discharge, n (%)	4,810 (61.8)	2,915 (60.1)	206 (61.5)	0.135
Age ≥ 75 years				
N	2,705	1,543	130	
In-hospital mortality, n (%)	248 (9.2)	140 (9.1)	19 (14.6)	0.105
mRS score ≤ 2 at discharge, n (%)	686 (25.4)	405 (26.2)	24 (18.5)	0.144
Clipping procedure subgroup				
N	6,347	3,679	214	
In-hospital mortality, n (%)	328 (5.2)	197 (5.4)	13 (6.1)	0.795
mRS score ≤ 2 at discharge, n (%)	3,299 (52.0)	1,887 (51.3)	105 (49.1)	0.597
Coiling procedure subgroup				
N	3,997	2,611	247	
In-hospital mortality, n (%)	289 (7.2)	164 (6.3)	47 (19.0)	< 0.001
mRS score ≤ 2 at discharge, n (%)	2134 (53.4)	1384 (53.0)	123 (49.8)	0.542
Concomitant medication of cilostazol				
N	5041	3209	108	
In-hospital mortality, n (%)	204 (4.0)	143 (4.5)	8 (7.4)	0.174
mRS score ≤ 2 at discharge, n (%)	2782 (55.2)	1774 (55.3)	58 (53.7)	0.948
Non-concomitant medication of cilostazol				
N	5443	3188	357	
In-hospital mortality, n (%)	422 (7.8)	223 (7.0)	52 (14.6)	< 0.001
mRS score ≤ 2 at discharge, n (%)	2714 (49.9)	1546 (48.5)	172 (48.2)	0.428

F group: fasudil hydrochloride, FO group: combination of fasudil hydrochloride and ozagrel sodium, O group: ozagrel sodium

mRS: modified Rankin Scale

**Table 4:** Effect of treatment drugs on in-hospital mortality of patients with subarachnoid hemorrhage—univariable and multivariable analysis results

		Univariable analysis			Multivariable analysis		
		Odds ratio	95% CI	p-value	Odds ratio	95% CI	p-value
In-hospital mortality	F group	Reference			Reference		
	FO group	0.95	0.83–1.08	0.424	0.94	0.81–1.08	0.355
	O group	2.33	1.76–3.10	< 0.001	2.05	1.50–2.80	< 0.001
Age	< 75 years	Reference			Reference		
	≥ 75 years	1.96	1.72–2.23	< 0.001	2.00	1.74–2.31	< 0.001
Sex	Male				Reference		
	Female	0.93	0.82–1.07	0.313	0.84	0.73–0.97	0.021
Treatment modality	Clipping	Reference			Reference		
	Coiling	1.41	1.24–1.59	< 0.001	1.31	1.11–1.53	< 0.001
	Clipping and coiling	1.12	0.66–1.89	0.686	1.04	0.59–1.83	0.880
Starting time of treatment	Day 1	Reference			Reference		
	Day 2	0.56	0.43–0.73	< 0.001	0.65	0.49–0.87	0.004
	Day 3	0.43	0.32–0.56	< 0.001	0.52	0.38–0.70	< 0.001
	Day ≥ 4	0.51	0.39–0.67	< 0.001	0.51	0.38–0.69	< 0.001
Ambulance use		1.84	1.47–2.32	< 0.001	1.19	0.93–1.52	0.158
Time between SAH and admission	Under 3 days	Reference			Reference		
	4–7 days	0.46	0.26–0.75	0.004	0.74	0.43–1.27	0.281
ICU admission		1.06	0.93–1.20	0.388	0.93	0.81–1.06	0.271
Artificial ventilation		6.14	5.05–7.54	< 0.001	4.46	3.61–5.51	< 0.001
Hospital case volume quartiles, case/4 years	1–7	Reference			Reference		
	8–17	0.85	0.62–1.15	0.284	0.74	0.53–1.02	0.067
	18–33	0.66	0.49–0.88	0.005	0.56	0.41–0.77	< 0.001
	≥ 34	0.65	0.50–0.86	0.002	0.50	0.37–0.67	< 0.001
JCS score at admission	0				Reference		
	1-digit code	1.51	1.16–1.97	0.002	1.30	0.99–1.72	0.058
	2-digit code	2.06	1.58–2.67	< 0.001	1.49	1.13–1.96	0.004
	3-digit code	5.72	4.53–7.24	< 0.001	3.15	2.45–4.05	< 0.001
mRS score at admission	0	Reference			Reference		
	1	1.09	0.90–1.33	0.371	0.96	0.78–1.18	0.688
	2	1.05	0.77–1.45	0.744	1.00	0.72–1.40	0.997
	3	1.46	1.03–2.08	0.036	1.38	0.95–2.00	0.096
	4	1.43	1.02–2.01	0.038	1.31	0.91–1.87	0.143
	5	2.45	1.95–3.07	< 0.001	1.49	1.17–1.89	0.001
Comorbidities							
Charlson Comorbidity Index	≥ 1	1.06	0.93–1.21	0.356	0.96	0.83–1.12	0.614
Hypertension		0.71	0.63–0.81	< 0.001	0.71	0.63–0.81	< 0.001
Diabetes		1.17	0.95–1.42	0.125	1.12	0.90–1.39	0.322
Hyperlipidemia		0.69	0.55–0.85	0.001	1.06	0.84–1.34	0.608
Cerebral infarction		0.86	0.66–1.12	0.277	0.90	0.68–1.20	0.468
Cerebral hemorrhage		2.33	1.78–3.02	< 0.001	1.43	1.07–1.90	0.016

F group: fasudil hydrochloride, FO group: combination of fasudil hydrochloride and ozagrel sodium, O group: ozagrel sodium

CI: confidence interval, ICU: intensive care unit, JCS: Japan Coma Scale, mRS: modified Rankin Scale, SAH: subarachnoid hemorrhage

F group values were used for reference

Multivariable factors for analysis: age, sex, treatment procedure for SAH, Japan Coma Scale score on admission, mRS score before onset of stroke, days from onset of subarachnoid hemorrhage to admission, starting time of treatment, ambulance use, Charlson Comorbidity Index, presence of comorbidities, concomitant medications, use of a specific intensive care unit, use of mechanical ventilation, and hospital case volume

**Table 5:** Effects of treatment drugs on good clinical outcome (mRS score ≤ 2 at discharge) among patients with subarachnoid hemorrhage—univariable and multivariable analysis results

		Univariable analysis			Multivariable analysis		
		Odds ratio	95% CI	p-value	Odds ratio	95% CI	p-value
mRS score ≤ 2 at discharge	F group	Reference			Reference		
	FO group	0.98	0.92–1.04	0.484	0.93	0.86–1.00	0.054
	O group	0.89	0.74–1.07	0.211	0.95	0.76–1.18	0.625
Age	< 75 years	Reference			Reference		
	≥ 75 years	0.22	0.20–0.23	< 0.001	0.18	0.17–0.20	< 0.001
Sex	Male	Reference			Reference		
	Female	0.78	0.73–0.83	< 0.001	1.06	0.98–1.15	0.117
Treatment modality	Clipping	Reference			Reference		
	Coiling	1.06	1.00–1.13	0.050	1.14	1.05–1.25	0.003
	Clipping and coiling	0.79	0.62–1.01	0.062	0.75	0.56–1.00	0.052
Starting time of treatment	Day 1	Reference			Reference		
	Day 2	1.08	0.92–1.27	0.361	1.13	0.93–1.38	0.213
	Day 3	1.03	0.88–1.22	0.695	1.08	0.89–1.32	0.438
	Day ≥ 4	0.80	0.68–0.94	0.006	0.89	0.73–1.08	0.241
Ambulance use		0.47	0.42–0.51	< 0.001	0.71	0.64–0.80	< 0.001
Time between SAH and admission	Under 3 days	Reference			Reference		
	4–7 days	1.75	1.46–2.11	< 0.001	0.97	0.78–1.20	0.757
ICU admission		0.95	0.90–1.01	0.091	0.99	0.92–1.06	0.770
Artificial ventilation		0.36	0.33–0.38	< 0.001	0.49	0.46–0.53	< 0.001
Hospital case volume quartiles, case/4 years	1–7	Reference			Reference		
	8–17	1.14	0.97–1.35	0.117	1.38	1.13–1.69	0.001
	18–33	1.24	1.05–1.45	0.008	1.52	1.26–1.83	< 0.001
	≥ 34	1.16	1.00–1.35	0.053	1.55	1.29–1.85	< 0.001
JCS score at admission	0	Reference			Reference		
	1-digit code	0.47	0.43–0.52	< 0.001	0.55	0.49–0.61	< 0.001
	2-digit code	0.32	0.29–0.35	< 0.001	0.40	0.36–0.45	< 0.001
	3-digit code	0.11	0.10–0.12	< 0.001	0.14	0.13–0.16	< 0.001
mRS score at admission	0	Reference					
	1	0.72	0.66–0.79	< 0.001	0.90	0.80–1.00	0.054
	2	0.64	0.55–0.75	< 0.001	0.70	0.59–0.84	< 0.001
	3	0.40	0.33–0.49	< 0.001	0.43	0.34–0.55	< 0.001
	4	0.53	0.44–0.64	< 0.001	0.63	0.50–0.77	< 0.001
	5	0.28	0.24–0.34	< 0.001	0.44	0.37–0.54	< 0.001
Comorbidities							
Charlson Comorbidity Index	≥ 1	0.86	0.81–0.92	< 0.001	1.01	0.93–1.09	0.846
Hypertension		1.05	0.99–1.11	0.117	1.08	1.00–1.15	0.042
Diabetes		0.59	0.53–0.65	< 0.001	0.61	0.54–0.70	< 0.001
Hyperlipidemia		1.37	1.25–1.51	< 0.001	1.14	1.02–1.28	0.019
Cerebral infarction		1.10	0.98–1.24	0.121	1.13	0.97–1.31	0.105
Cerebral hemorrhage		0.31	0.25–0.38	< 0.001	0.48	0.38–0.60	< 0.001

F group: fasudil hydrochloride, FO group: combination of fasudil hydrochloride and ozagrel sodium, O group: ozagrel sodium

CI: confidence interval, ICU: intensive care unit, JCS: Japan Coma Scale, mRS: modified Rankin Scale, SAH: subarachnoid hemorrhage

F group values were used for reference

Multivariable factors for analysis: age, sex, treatment procedure for SAH, Japan Coma Scale score on admission, mRS score before onset of stroke, days from onset of subarachnoid hemorrhage to admission, starting time of treatment, ambulance use, Charlson Comorbidity Index, presence of comorbidities, concomitant medications, use of a specific intensive care unit, use of mechanical ventilation, and hospital case volume

Patients who underwent both clipping and coiling were excluded.

Subgroup analyses were performed to assess whether age and treatment procedures affect clinical outcomes after SAH (Tables 3 and 6; see Additional files 6 and 7). We also performed subgroup analyses for cilostazol to investigate the impact of concomitant medications (Tables 3 and 6; see Additional file 8). With regard to in-hospital mortality, the adjusted OR for ages < 75 years was 0.87 in the FO group (95% CI: 0.73–1.04, *p* = 0.133) and 2.25 in the O group (95% CI: 1.53–3.31, *p* < 0.001) and for ages ≥ 75 years was 1.02 in the FO group (95% CI: 0.81–1.29, *p* = 0.839) and 1.67 in the O group (95% CI: 0.97–2.86, *p* = 0.065). With regard to the proportion of patients with mRS score ≤ 2 at discharge, the adjusted OR for ages < 75 years was 0.92 (95% CI: 0.84–1.00, *p* = 0.047) and 1.07 (95% CI: 0.82–1.39, *p* = 0.622) and for ages ≥ 75 years was 0.95 (95% CI: 0.81–1.11, *p* = 0.506) and 0.63 (95% CI: 0.39–1.03, *p* = 0.064) in the FO and O groups, respectively. Therefore, the results obtained for each age subgroup were similar to the overall results.

**Table 6:** Effects of treatment drugs on in-hospital mortality or good clinical outcome (mRS score ≤ 2 at discharge) among patients with subarachnoid hemorrhage—multivariable analysis subgroup (age, treatment procedure, and concomitant medication of cilostazol) results

		< 75 years			≥ 75 years		
		Odds ratio	95% CI	p-value	Odds ratio	95% CI	p-value
In-hospital mortality	F group	Reference			Reference		
	FO group	0.87	0.73–1.04	0.133	1.02	0.81–1.29	0.839
	O group	2.25	1.53–3.31	< 0.001	1.67	0.97–2.86	0.065
mRS score ≤ 2 at discharge	F group	Reference			Reference		
	FO group	0.92	0.84–1.00	0.047	0.95	0.81–1.11	0.506
	O group	1.07	0.82–1.39	0.622	0.63	0.39–1.03	0.064
		Clipping			Coiling		
		Odds ratio	95% CI	p-value	Odds ratio	95% CI	p-value
In-hospital mortality	F group	Reference			Reference		
	FO group	1.04	0.86–1.26	0.686	0.83	0.67–1.03	0.084
	O group	1.11	0.61–2.04	0.731	2.75	1.86–4.06	< 0.001
mRS score ≤ 2 at discharge	F group	Reference			Reference		
	FO group	0.95	0.86–1.04	0.260	0.92	0.81–1.03	0.160
	O group	1.08	0.78–1.49	0.652	0.82	0.60–1.11	0.202
		Concomitant medication of cilostazol			Non-concomitant medication of cilostazol		
		Odds ratio	95% CI	p-value	Odds ratio	95% CI	p-value
In-hospital mortality	F group	Reference			Reference		
	FO group	1.13	0.90–1.43	0.290	0.85	0.71–1.02	0.075
	O group	1.85	0.84–4.04	0.124	1.98	1.39–2.81	< 0.001
mRS score ≤ 2 at discharge	F group	Reference			Reference		
	FO group	0.99	0.89–1.11	0.887	0.84	0.75–0.93	0.001
	O group	1.00	0.63–1.60	0.996	0.92	0.70–1.20	0.539

F group: fasudil hydrochloride, FO group: combination of fasudil hydrochloride and ozagrel sodium, O group: ozagrel sodium

CI: confidence interval, mRS: modified Rankin Scale, SAH: subarachnoid hemorrhage

F group data were used for reference

Multivariable factors for subgroup analysis by age: sex, treatment procedure for SAH (clipping, coiling), Japan Coma Scale score on admission, mRS score before onset of stroke, days from onset of subarachnoid hemorrhage to admission (within the 3rd day of onset, within the 4th day of onset to the 7th day of onset), starting time of treatment, ambulance use, Charlson Comorbidity Index (0, ≥ 1), presence of comorbidities (hypertension, diabetes mellitus, dyslipidemia, cerebral infarction, cerebral hemorrhage), concomitant medications (cilostazol, statins, edaravone, antihypertensive drug, antiplatelet drug), use of a specific intensive care unit, use of mechanical ventilation, and hospital case volume

Multivariable factors for subgroup analysis by treatment procedure: sex, age, Japan Coma Scale score on admission, mRS score before onset of stroke, days from onset of subarachnoid hemorrhage to admission (within the 3rd day of onset, within the 4th day of onset to the 7th day of onset), starting time of treatment, ambulance use, Charlson Comorbidity Index (0, ≥ 1), presence of comorbidities (hypertension, diabetes mellitus, dyslipidemia, cerebral infarction, cerebral hemorrhage), concomitant medications (cilostazol, statins, edaravone, antihypertensive drug, antiplatelet drug), use of a specific intensive care unit, use of mechanical ventilation and hospital case volume

Multivariable factors for subgroup analysis by concomitant medication of cilostazol: sex, age, treatment procedure for SAH (clipping, coiling), Japan Coma Scale score on admission, mRS score before onset of stroke, days from onset of subarachnoid hemorrhage to admission (within the 3rd day of onset, within the 4th day of onset to the 7th day of onset), starting time of treatment, ambulance use, Charlson Comorbidity Index (0, ≥ 1), presence of comorbidities (hypertension, diabetes mellitus, dyslipidemia, cerebral infarction, cerebral hemorrhage), concomitant medications (statins, edaravone, antihypertensive drug, antiplatelet drug), use of a specific intensive care unit, use of mechanical ventilation and hospital case volume

For the subgroup analysis based on treatment procedures, the adjusted OR for in-hospital mortality for clipping was 1.04 in the FO group (95% CI: 0.86–1.26, *p* = 0.686) and 1.11 in the O group (95% CI: 0.61–2.04, *p* = 0.731) and for coiling was 0.83 in the FO group (95% CI: 0.67–1.03, *p* = 0.084) and 2.75 in the O group (95% CI: 1.86–4.06, *p* < 0.001). Regarding the proportion of patients with mRS score ≤ 2 at discharge, the adjusted OR for clipping was 0.95 (95% CI: 0.86–1.04, *p* = 0.260) and 1.08 (95% CI: 0.78–1.49, *p* = 0.652) and for coiling was 0.92 (95% CI: 0.81–1.03, *p* = 0.160) and 0.82 (95% CI: 0.60–1.11, *p* = 0.202) in the FO and O groups, respectively. Therefore, the in-hospital mortality when coiling was used as the treatment procedure was greater than that of clipping.

For the subgroup analysis based on concomitant medication of cilostazol, the adjusted OR for in-hospital mortality for concomitant medication of cilostazol was 1.13 in the FO group (95% CI: 0.90–1.43, *p* = 0.290) and 1.85 in the O group (95% CI: 0.84–4.04, 0.124) and for non-concomitant medication of cilostazol, it was 0.85 in the FO group (95% CI: 0.71–1.02, *p* = 0.075) and 1.98 in the O group (95% CI: 1.39–2.81, *p* < 0.001). For the proportion of patients with an mRS score ≤ 2 at discharge, the adjusted OR for concomitant medication of cilostazol was 0.99 (95% CI: 0.89–1.11, *p* = 0.887) and 1.00 (95% CI: 0.63–1.60, *p* = 0.996), and that for non-concomitant medication of cilostazol was 0.84 (95% CI: 0.75–0.93, *p* = 0.001) and 0.92 (95% CI: 0.70–1.20, *p* = 0.539) in the FO and O groups, respectively. Therefore, the in-hospital mortality when cilostazol was not used as the concomitant medication was greater than that when the concomitant medication was cilostazol.

## Discussion

This cross-sectional study involved 17,346 patients with aSAH who underwent clipping or coiling procedures and subsequently received fasudil hydrochloride (F group), ozagrel sodium (O group), or a combination of both (FO group). The primary endpoint of in-hospital mortality and the secondary endpoint of mRS ≤ 2 at discharge did not differ between the F group and the FO group. This finding was like that reported by Suzuki et al. [10], that the combination of fasudil hydrochloride and ozagrel sodium was well tolerated but did not provide superior efficacy to that of fasudil monotherapy.

When comparing the FO and O groups, no difference was found in the proportion of mRS ≤ 2 at discharge. However, in-hospital mortality tended to decrease in the FO group. Using symptomatic cerebral vasospasm and low-density areas in the brain as indicators, Nakashima et al. reported a comparative study of ozagrel sodium monotherapy and ozagrel sodium and fasudil hydrochloride combination therapy [11]. In this report, it is considered that the combination therapy of ozagrel sodium and fasudil hydrochloride is more effective than using only ozagrel sodium in treating patients at risk of vasospasm after aneurysmal subarachnoid hemorrhage. Despite the different outcome measures, our results suggest that the combination of ozagrel sodium and fasudil hydrochloride has a prognostic impact compared with ozagrel monotherapy.

The main pharmacological effect of fasudil hydrochloride is vasodilation due to the inhibition of rho kinase; ozagrel sodium primarily inhibits platelet aggregation and vascular smooth muscle contraction due to the inhibition of thromboxane synthase. In dogs, fasudil hydrochloride, but not ozagrel sodium, has been reported to markedly ameliorate arterial stenosis during the chronic phase of cerebral vasospasm [21]. These findings suggest that thromboxane A2 synthase (TXA2) is not involved in maintaining the chronic phase of cerebral vasospasm after SAH and that protein kinases, particularly myosin light chain kinase and protein kinase C, are involved in the pathology of arterial stenosis during the chronic phase of cerebral vasospasm [21]. The cytoprotective effects of fasudil hydrochloride on delayed neuronal death in gerbil hippocampi were compared with those of nimodipine, a calcium channel blocker, and ozagrel sodium, a TXA2 inhibitor; in the study, fasudil hydrochloride significantly reduced the loss of nerve cells in the ischemic control group, whereas nimodipine and ozagrel sodium did not [22]. These results suggest that ozagrel sodium exerts a synergistic effect by inhibiting the contraction of vascular smooth muscle and arterial stenosis in the chronic phase of cerebral vasospasm is inhibited by concomitant fasudil hydrochloride administration. Moreover, when comparing the F group and the O group, in-hospital mortality tended to be higher in the O group, and the proportion of discharge mRS ≤ 2 tended to be lower in the O group. The proportion of patients without moderate and severe cerebral vasospasm after single-agent fasudil hydrochloride administration was 62% [6], and the proportion of patients without symptomatic cerebral vasospasm after single-agent ozagrel sodium administration was reported to be 54% [7]. However, there were no reports of direct comparisons between fasudil hydrochloride monotherapy and ozagrel sodium monotherapy, suggesting that the prognostic impact of both drugs should be further investigated.

As shown in Table 1, the starting time of treatment tended to be slightly later in the FO and O groups than in the F group. Therefore, the multivariate analysis was performed by incorporating the starting time of treatment as a multivariate analysis factor, and there was no effect on worse prognosis for small differences in the starting time of treatment.

Notably, a reminder statement regarding the risk of cerebral hemorrhage is provided in the “Important Precautions” section of the package insert for both fasudil hydrochloride and ozagrel sodium. Consequently, we evaluated cerebral hemorrhage as an event occurring during hospitalization, and its incidence was not significantly different between the three groups.

In the present study, we conducted a subgroup analysis to investigate the effects of age; our findings showed that mortality was higher and the proportion of mRS score ≤ 2 was lower in the ≥ 75-year group than in the < 75-year group. Age has also been reported to be an independent risk factor for poor functional outcomes due to aneurysm rupture [23], and differences in functional outcomes at discharge have been shown between non-elderly and elderly groups [16]. Although the effect of treatment with cerebral vasospastic agents on in-hospital mortality did not differ between the < 75-year and ≥ 75-year groups, the results of the present study are consistent with those of these previous studies. As the in-hospital mortality OR in the ozagrel sodium group was greater for ages < 75 years than that for ages ≥ 75 years, combination therapy with ozagrel sodium and fasudil hydrochloride, rather than ozagrel monotherapy, should be considered more appropriate for patients aged < 75 years.

This study showed that coiling was performed more often in the O group than in the other groups (Table 3), and subgroup analysis revealed that clipping had a better effect on clinical outcomes than coiling, which was also one of the factors associated with a higher in-hospital mortality. Kurogi et al. reported that patients with ruptured aneurysms who received coiling as a rebleeding prevention procedure showed a significantly higher proportion of in-hospital mortality than patients who received clipping (coiling, 12.4% vs. clipping, 8.7%; OR, 1.3) [24]. Similar to the report by Kurogi et al., the higher proportion of coiling procedures in the O group was considered a factor associated with higher proportion of in-hospital mortality.

This study showed that for the aneurysm location, the ratio of posterior circulation was higher in the O group (basilar artery: F group 3.8%, FO group 3.9%, O group 6.5%; vertebral artery: F group 6.8%, FO group 6.5%, O group 9.2%, Table 2). In accordance with these results, Göttsche et al. reported that patients with an aneurysm of the posterior circulation had a worse functional outcome than those with an anterior circulation aneurysm [25]. We also showed that the O group had a lower percentage of individuals with a high case volume (hospital case volume quartiles, cases/4 years: F group 55.7%, FO group 57.2%, O group 41.3%, Table 2). Correspondingly, Kurogi et al. reported that in patients who underwent coiling, a high case volume (> 9 cases/year) was associated with reduced in-hospital mortality and short-term adverse outcomes [26]. Therefore, aneurysm location and hospital case volume were considered to be factors associated with the higher in-hospital mortality in the O group.

In addition to fasudil hydrochloride and ozagrel sodium, various other drugs have been used to treat cerebral vasospasm [2].

This study showed that the concomitant use of cilostazol, an antiplatelet agent, was lower in the O group than in the F and the FO groups (F group 48.1%, FO group 50.2%, O group 23.2%, Table 2). For the results of subgroup analysis, the effect on in-hospital mortality was also greater in the absence of cilostazol than in the presence of cilostazol in the treatment procedure (OR of in-hospital mortality in the O group: concomitant medication of cilostazol 1.85, non-concomitant medication of cilostazol 1.98, Table 6). In accordance with these findings, a systematic review examining the effects of cilostazol on cerebral vasospasm showed significantly lower incidences of symptomatic cerebral vasospasm, severe cerebral vasospasm, cerebral infarction, and unfavorable outcomes in patients who received cilostazol than in those who did not [27]. Therefore, the low ratio of the concomitant use of cilostazol in the O group was considered one of the contributing factors for the high proportion of in-hospital mortality in the O group.

Our results also indicate that the use of statins was lower in the O group than in the F and FO groups. A systematic review examining the effects of statins on cerebral vasospasm indicated that statins reduce the incidence of delayed ischemic neurological deficits due to cerebral vasospasm, but do not significantly improve functional outcomes [28]. Therefore, further investigations are required to elucidate the effects of concomitant statins on the proportion of in-hospital mortality in patients with SAH.

This study has several limitations. First, in the subgroup analysis to examine the effects of age and treatment procedure, in-hospital mortality and proportion of mRS ≤ 2 at discharge in the subgroups were similar to those in the overall analysis. However, in the O group, the adjusted OR was higher in the < 75-year subgroup and the coiling subgroup, and further studies are deemed necessary to clarify these trends. Second, the DPC database comprises data from during the hospital stay. Thus, we were unable to assess long-term outcomes after discharge and adopted the mRS score at discharge as an endpoint. Third, the DPC database does not include the Hunt-Hess scale and World Federation of Neurological Surgeons grade data, which are indicative of the severity of SAH. Therefore, SAH severity was adjusted according to the JCS score. Fourth, the DPC database does not include information regarding the presence or absence of cerebral vasospasm; consequently, efficacy in treating cerebral vasospasm could not be evaluated. As fasudil hydrochloride and ozagrel sodium are therapeutic agents for cerebral vasospasm, a more detailed discussion may be possible by evaluating its incidence. Fifth, the inability of the DPC database to capture patients’ multiple-hospital visits limits the adequate collection of data on concomitant medications and comorbidities. Lastly, as the DPC database covers many stroke treatment hospitals in Japan, our findings can be generalized to Japanese institutions. However, nimodipine use has become the standard of care in most countries in the USA and EU. Therefore, our findings may not be generalizable to these or other countries with distinct healthcare resources and systems.

## Conclusions

Fasudil hydrochloride and ozagrel sodium had different mechanisms of action, suggesting a synergistic effect of combination therapy. However, a comparison of fasudil hydrochloride monotherapy and combination therapy of fasudil hydrochloride and ozagrel sodium showed no difference in the prognostic effect. Therefore, it was suggested that fasudil hydrochloride monotherapy may be sufficient. Future studies should be conducted to further investigate the therapeutic effects of ozagrel sodium and fasudil hydrochloride alone, in combination, or combined with other agents.

### Electronic supplementary material

Below is the link to the electronic supplementary material.

Supplementary Material 1

Supplementary Material 2

Supplementary Material 3

Supplementary Material 4

Supplementary Material 5

Supplementary Material 6

Supplementary Material 7

Supplementary Material 8

## Data Availability

The data that support the findings of this study are not openly available due to reasons of sensitivity and are available from the corresponding author upon reasonable request.

## Abbreviations

SD: Standard deviation
F: Fasudil hydrochloride
O: Ozagrel sodium
FO: Combination of fasudil hydrochloride and ozagrel sodium
OR: Odds ratio
CI: Confidence interval
mRS: Modified Rankin Scale
SAH: Subarachnoid hemorrhage
TXA2: Thromboxane A2
DPC: Diagnosis procedure combination
JCS: Japan Coma Scale

## References

[1] Macdonald RL, Schweizer TA. Spontaneous subarachnoid haemorrhage. Lancet. 2017;389:655–66. 10.1016/S0140-6736(16)30668-7. 27637674 10.1016/S0140-6736(16)30668-7

[2] Daou BJ, Koduri S, Thompson BG, Chaudhary N, Pandey AS. Clinical and experimental aspects of aneurysmal subarachnoid hemorrhage, CNS neurosci. Ther. 2019;25:1096–112. 10.1111/cns.13222. 10.1111/cns.13222 PMC6776745 31583833

[3] Hoh BL, Ko NU, Amin-Hanjani S, Chou SHY, Cruz-Flores S, Dangayach NS, et al. 2023 Guideline for the management of patients with aneurysmal subarachnoid hemorrhage: a guideline from the American Heart Association/American Stroke Association. Stroke. 2023;54:e314–70. 10.1161/STR.0000000000000436. 37212182 10.1161/STR.0000000000000436

[4] Toyoda K, Yoshimura S, Nakai M, Koga M, Sasahara Y, Sonoda K, et al. Japan Stroke Data Bank Investigators, twenty-year change in severity and outcome of ischemic and hemorrhagic strokes. JAMA Neurol. 2022;79:61–9. 10.1001/jamaneurol.2021.4346. 34870689 10.1001/jamaneurol.2021.4346 PMC8649912

[5] Steiner T, Juvela S, Unterberg A, Jung C, Forsting M, Rinkel G. European Stroke Organization, European Stroke Organization Guidelines for the management of intracranial aneurysms and subarachnoid haemorrhage. Cerebrovasc Dis. 2013;35:93–112. 10.1159/000346087. 23406828 10.1159/000346087

[6] Shibuya M, Suzuki Y, Sugita K, Saito I, Sasaki T, Takakura K, et al. Effect of AT877 on cerebral vasospasm after aneurysmal subarachnoid hemorrhage. Results of a prospective placebo-controlled double‐blind trial. J Neurosurg. 1992;76:571–7. 10.3171/jns.1992.76.4.0571. 1545249 10.3171/jns.1992.76.4.0571

[7] Tokiyoshi K, Ohnishi T, Nii Y. Efficacy and toxicity of thromboxane synthetase inhibitor for cerebral vasospasm after subarachnoid hemorrhage. Surg Neurol. 1991;36:112–8. 10.1016/0090-3019(91)90228-2. 1891755 10.1016/0090-3019(91)90228-2

[8] Miyamoto S, Ogasawara K, Kuroda S, Itabashi R, Toyoda K, Itoh Y, et al. Int J Stroke. 2022;17:1039–49. 10.1177/17474930221090347. Committee for Stroke Guideline 2021, the Committee for Stroke Guideline 2021, the Japan Stroke Society, Japan Stroke Society Guideline 2021 for the Treatment of Stroke. 10.1177/17474930221090347 PMC9615334 35443847

[9] Koumura A, Hamanaka J, Kawasaki K, Tsuruma K, Shimazawa M, Hozumi I, et al. Fasudil and ozagrel in combination show neuroprotective effects on cerebral infarction after murine middle cerebral artery occlusion. J Pharmacol Exp Ther. 2011;338:337–44. 10.1124/jpet.110.177675. 21493751 10.1124/jpet.110.177675

[10] Suzuki Y, Shibuya M, Satoh S, Sugiyama H, Seto M, Takakura K. Safety and efficacy of fasudil monotherapy and fasudil-ozagrel combination therapy in patients with subarachnoid hemorrhage: sub-analysis of the post-marketing surveillance study. Neurol Med Chir (Tokyo). 2008;48:241–7. 10.2176/nmc.48.241. discussion 247–248. 18574328 10.2176/nmc.48.241

[11] Nakashima S, Tabuchi K, Shimokawa S, Fukuyama K, Mineta T, Abe M. Combination therapy of fasudil hydrochloride and ozagrel sodium for cerebral vasospasm following aneurysmal subarachnoid hemorrhage. Neurol Med Chir (Tokyo). 1998;38:805–10. 10.2176/nmc.38.805. discussion 810. 10063353 10.2176/nmc.38.805

[12] Yasunaga H, Matsui H, Horiguchi H, Fushimi K, Matsuda S. Clinical epidemiology and health services research using the diagnosis Procedure Combination database in Japan, Asian Pac. J Dis Manag. 2015;7:19–24. 10.7223/apjdm.7.19. 10.7223/apjdm.7.19

[13] Hayashida K, Murakami G, Matsuda S, Fushimi K. History and profile of diagnosis Procedure Combination (DPC): development of a real data collection system for acute inpatient care in Japan. J Epidemiol. 2021;31:1–11. 10.2188/jea.JE20200288. 33012777 10.2188/jea.JE20200288 PMC7738645

[14] Shigematsu K, Nakano H, Watanabe Y. The eye response test alone is sufficient to predict stroke outcome–reintroduction of Japan Coma Scale: a cohort study. BMJ (Open). 2013;3:e002736. 10.1136/bmjopen-2013-002736. 23633419 10.1136/bmjopen-2013-002736 PMC3641437

[15] Hsieh MT, Huang KC, Hsieh CY, Tsai TT, Chen LC, Sung SF. Validation of ICD-10-CM diagnosis codes for identification of patients with acute hemorrhagic stroke in a national health insurance claims database. Clin Epidemiol. 2021;13:43–51. 10.2147/CLEP.S288518. 33469381 10.2147/CLEP.S288518 PMC7813455

[16] Ido K, Kurogi R, Kurogi A, Nishimura K, Arimura K, Nishimura A, et al. Effect of treatment modality and cerebral vasospasm agent on patient outcomes after aneurysmal subarachnoid hemorrhage in the elderly aged 75 years and older. PLoS ONE. 2020;15:e0230953. 10.1371/journal.pone.0230953. 32271814 10.1371/journal.pone.0230953 PMC7145106

[17] Nakajima M, Okada Y, Sonoo T, Goto T. Development and validation of a novel method for converting the Japan Coma Scale to Glasgow Coma Scale. J Epidemiol. 2023;33:531–5. 10.2188/jea.JE20220147. 35851565 10.2188/jea.JE20220147 PMC10483104

[18] Ohkuma H, Shimamura N, Naraoka M, Katagai T. Aneurysmal subarachnoid hemorrhage in the elderly over age 75: a systematic review. Neurol Med Chir (Tokyo). 2017;57:575–83. 10.2176/nmc.ra.2017-0057. 28835583 10.2176/nmc.ra.2017-0057 PMC5709710

[19] Kada A, Nishimura K, Nakagawara J, Ogasawara K, Ono J, Shiokawa Y, et al. Development and validation of a score for evaluating comprehensive stroke care capabilities: J-ASPECT study. BMC Neurol. 2017;17:46. 10.1186/s12883-017-0815-4. 28241749 10.1186/s12883-017-0815-4 PMC5330137

[20] Quan H, Sundararajan V, Halfon P, Fong A, Burnand B, Luthi JC, et al. Coding algorithms for defining comorbidities in ICD-9-CM and ICD-10 administrative data, Med. Care. 2005;43:1130–9. 10.1097/01.mlr.0000182534.19832.83. 10.1097/01.mlr.0000182534.19832.83 16224307

[21] Toshima Y, Satoh S, Ikegaki I, Asano T, Suzuki Y, Shibuya M. Thromboxane A2 synthetase inhibitor failed to ameliorate the arterial narrowing during the chronic phase of cerebral vasospasm. Life Sci. 1997;61:1371–7. 10.1016/s0024-3205(97)00682-6. 9335226 10.1016/s0024-3205(97)00682-6

[22] Satoh S, Ikegaki I, Suzuki Y, Asano T, Shibuya M, Hidaka H. Neuroprotective properties of a protein kinase inhibitor against ischaemia-induced neuronal damage in rats and gerbils. Br J Pharmacol. 1996;118:1592–6. 10.1111/j.1476-5381.1996.tb15579.x. 8842419 10.1111/j.1476-5381.1996.tb15579.x PMC1909837

[23] Rinaldo L, Rabinstein AA, Lanzino G. Elderly age associated with poor functional outcome after rupture of anterior communicating artery aneurysms. J Clin Neurosci. 2016;34:108–11. 10.1016/j.jocn.2016.05.006. 27436764 10.1016/j.jocn.2016.05.006

[24] Kurogi R, Kada A, Nishimura K, Kamitani S, Nishimura A, Sayama T, et al. Effect of treatment modality on in-hospital outcome in patients with subarachnoid hemorrhage: a nationwide study in Japan (J-ASPECT Study). J Neurosurg. 2018;128:1318–26. 10.3171/2016.12.JNS161039. 28548595 10.3171/2016.12.JNS161039

[25] Göttsche J, Piffko A, Pantel TF, Westphal M, Dührsen L, Czorlich P, Sauvigny T. Aneurysm location affects clinical course and mortality in patients with subarachnoid hemorrhage. Front Neurol. 2022;13:846066. 10.3389/fneur.2022.846066. 35359650 10.3389/fneur.2022.846066 PMC8964037

[26] Kurogi R, Kada A, Ogasawara K, Kitazono T, Sakai N, Hashimoto Y, et al. Effects of case volume and comprehensive stroke center capabilities on patient outcomes of clipping and coiling for subarachnoid hemorrhage. J Neurosurg. 2020;134:929–39. 10.3171/2019.12.JNS192584. 32168489 10.3171/2019.12.JNS192584

[27] Niu PP, Yang G, Xing YQ, Guo ZN, Yang Y. Effect of cilostazol in patients with aneurysmal subarachnoid hemorrhage: a systematic review and meta-analysis. J Neurol Sci. 2014;336:146–51. 10.1016/j.jns.2013.10.027. 24211059 10.1016/j.jns.2013.10.027

[28] Kramer AH, Fletcher JJ. Statins in the management of patients with aneurysmal subarachnoid hemorrhage: a systematic review and meta-analysis. Neurocrit Care. 2010;12:285–96. 10.1007/s12028-009-9306-9. 19921470 10.1007/s12028-009-9306-9

