# Supplementary material for: Fasudil hydrochloride and ozagrel sodium combination therapy for patients with aneurysmal subarachnoid hemorrhage: a cross-sectional study using a nationwide inpatient database
Source: J Pharm Health Care Sci. 2024 Aug 13;10:49. doi: 10.1186/s40780-024-00370-w (PMC11321058; doi:10.1186/s40780-024-00370-w)
Supplement: Supplementary file 1 — Supplementary Material 1 [file 40780_2024_370_MOESM1_ESM.docx]

Additional file 5. Baseline characteristics of patients in the F, FO, O groups and non-administration of drugs (fasudil hydrochloride and ozagrel sodium)

|  | F group  (n=10,484) | FO group  (n=6,397) | O group  (n=465) | Non-administration of fasudil hydrochloride and ozagrel sodium  (n=20,430) |
| --- | --- | --- | --- | --- |
| Age, years, mean (SD) | 63.6 (14.4) | 63.0 (14.4) | 64.4 (14.8) | 63.8 (14.5) |
| Age group |  |  |  |  |
| < 75 years, n (%) | 7779 (74.2) | 4854 (75.9) | 335 (72.0) | 15005 (73.4) |
| ≥ 75 years, n (%) | 2705 (25.8) | 1543 (24.1) | 130 (28.0) | 5425 (26.6) |
| Sex, n (%) |  |  |  |  |
| Male | 3157 (30.1) | 1938 (30.3) | 160 (34.4) | 6194 (30.3) |
| Female | 7327 (69.9) | 4459 (69.7) | 305 (65.6) | 14236 (69.7) |
| Location of aneurysms, n (%) |  |  |  |  |
| ICA | 2921 (27.9) | 1856 (29.0) | 132 (28.4) | 5814 (28.5) |
| MCA | 2456 (23.4) | 1412 (22.1) | 83 (17.8) | 4551 (22.3) |
| AcomA | 2528 (24.1) | 1646 (25.7) | 113 (24.3) | 4749 (23.2) |
| PcomA | 160 (1.5) | 109 (1.7) | 13 (2.8) | 305 (1.5) |
| BA | 397 (3.8) | 250 (3.9) | 30 (6.5) | 954 (4.7) |
| VA | 713 (6.8) | 418 (6.5) | 43 (9.2) | 1583 (7.7) |
| Other | 1578 (15.1) | 854 (13.4) | 65 (14.0) | 3034 (14.9) |
| Treatment modality, n (%) |  |  |  |  |
| Clipping | 6347 (60.5) | 3679 (57.5) | 214 (46.0) | 11136 (54.5) |
| Coiling | 3997 (38.1) | 2611 (40.8) | 247 (53.1) | 9036 (44.2) |
| Clipping and coiling | 140 (1.3) | 107 (1.7) | 4 (0.9) | 258 (1.3) |
| Ambulance use, n (%) | 9069 (86.5) | 5593 (87.4) | 383 (82.4) | 17623 (86.3) |
| Days from onset of SAH to admission, n (%) |  |  |  |  |
| ≤ 3 days | 10155 (96.9) | 6248 (97.7) | 442 (95.1) | 19647 (96.2) |
| 4-7 days | 329 (3.1) | 149 (2.3) | 23 (4.9) | 783 (3.8) |
| ICU admission, n (%) | 4842 (46.2) | 2921 (45.7) | 216 (46.5) | 1203 (5.9) |
| Artificial ventilation, n (%) | 6359 (60.7) | 3928 (61.4) | 243 (52.3) | 87 (0.4) |
| Length of hospital stay (SD) | 48.1 (46.9) | 48.7 (40.0) | 45.4 (39.3) | 1550 (7.6) |
| Hospital case volume quartiles, case/4 years, n (%) |  |  |  |  |
| 1-7 | 428 (4.1) | 247 (3.9) | 35 (7.5) | 224 (1.1) |
| 8-17 | 1278 (12.2) | 972 (15.2) | 119 (25.6) | 695 (3.4) |
| 18-33 | 2938 (29.0) | 1516 (23.7) | 119 (25.6) | 1846 (9.0) |
| ≥ 34 | 5840 (55.7) | 3662 (57.2) | 192 (41.3) | 17665 (86.5) |
| JCS score at admission, n (%) |  |  |  |  |
| 0 | 2041(19.5) | 1271 (19.9) | 104 (22.4) | 3991 (19.5) |
| 1-digit code | 2897 (27.6) | 1768 (27.6) | 129 (27.7) | 5408 (26.5) |
| 2-digit code | 2496 (23.8) | 1593 (24.9) | 106 (22.8) | 4677 (22.9) |
| 3-digit code | 3050 (29.1) | 1765 (27.6) | 126 (27.1) | 6354 (31.1) |
| GCS, n (%) |  |  |  |  |
| 15 | 3788 (36.1) | 2308 (36.1) | 187 (40.2) | 7269 (35.6) |
| 14 | 614 (5.9) | 363 (5.7) | 23 (4.9) | 1120 (5.5) |
| 13 | 536 (5.1) | 368 (5.8) | 23 (4.9) | 1010 (4.9) |
| 12-7 | 3266 (31.2) | 2080 (32.5) | 135 (29.0) | 6208 (30.4) |
| 6-3 | 2280 (21.7) | 1278 (20.0) | 97 (20.9) | 4823 (23.6) |
| mRS score before onset of stroke, n (%) |  |  |  |  |
| 0 | 7810 (74.5) | 4767 (74.5) | 320 (68.8) | 15176 (74.3) |
| 1 | 1189 (11.3) | 792 (12.4) | 74 (15.9) | 2544 (12.5) |
| 2 | 431 (4.1) | 267 (4.2) | 32 (6.9) | 854 (4.2) |
| 3 | 286 (2.7) | 138 (2.2) | 14 (3.0) | 529 (2.6) |
| 4 | 310 (3.0) | 167 (2.6) | 6 (1.3) | 535 (2.6) |
| 5 | 458 (4.4) | 266 (4.2) | 19 (4.1) | 792 (3.9) |
| Charlson Comorbidity Index, n (%) |  |  |  |  |
| 0 | 6640 (63.3) | 4247 (66.4) | 310 (66.7) | 13554 (66.3) |
| ≥ 1 | 3844 (36.7) | 2150 (33.6) | 155 (33.3) | 6876 (33.7) |
| Comorbidities, n (%) |  |  |  |  |
| Hypertension | 5995 (57.2) | 3645 (57.0) | 287 (61.7) | 11308 (55.3) |
| Diabetes | 1027 (9.8) | 607 (9.5) | 45 (9.7) | 1977 (9.7) |
| Hyperlipidemia | 1237 (11.8) | 775 (12.1) | 43 (9.2) | 2253 (11.0) |
| Cerebral infarction | 617 (5.9) | 471 (7.4) | 38 (8.2) | 1206 (5.9) |
| Cerebral hemorrhage | 341 (3.3) | 170 (2.7) | 15 (3.2) | 813 (4.0) |
| Concomitant medication, n (%) |  |  |  |  |
| Cilostazol | 5041 (48.1) | 3209 (50.2) | 108 (23.2) | 356 (1.7) |
| Statins | 3976 (37.9) | 2558 (40.0) | 85 (18.3) | 475 (2.3) |
| Edaravone | 2961 (28.2) | 2208 (34.5) | 192 (41.3) | 627 (3.1) |
| Catecholamine | 778 (7.4) | 537 (8.4) | 51 (11.0) | 332 (1.6) |
| Antihypertensive drug | 9164 (87.4) | 5631 (88.0) | 347(74.6) | 2185 (10.7) |
| Antiplatelet drug | 2627 (25.1) | 1924 (30.1) | 179 (38.5) | 659 (3.2) |

F group: fasudil hydrochloride, FO group: combination of fasudil hydrochloride and ozagrel sodium, O group: ozagrel sodium.

AcomA: anterior communicating artery, BA: basilar artery, GCS: Glasgow Coma Scale, ICA: internal carotid artery, ICU: intensive care unit, JCS: Japan Coma Scale, MCA: middle cerebral artery, mRS: modified Rankin Scale, PcomA: posterior communicating artery, SD: standard deviation, VA: vertebral artery.

Concomitant medications: All medications were administered during hospitalization.

Antihypertensive drug: Antihypertensive agents (Aspirin, Dipyridamole, Ticlopidine, Clopidogrel, and Prasugrel) used for acute treatment after subarachnoid hemorrhage.
