# Supplementary material for: Fasudil hydrochloride and ozagrel sodium combination therapy for patients with aneurysmal subarachnoid hemorrhage: a cross-sectional study using a nationwide inpatient database
Source: J Pharm Health Care Sci. 2024 Aug 13;10:49. doi: 10.1186/s40780-024-00370-w (PMC11321058; doi:10.1186/s40780-024-00370-w)
Supplement: Supplementary file 2 — Supplementary Material 2 [file 40780_2024_370_MOESM2_ESM.docx]

Additional file 1. Definition of drugs (Receipt computed code)

| Drug | Receipt computed code |
| --- | --- |
| Fasudil hydrochloride | 620005150, 622804001 |
| Ozagrel sodium | 620002253, 620002254, 620002913, 620002914, 620002915, 620002925  620002926, 620002933, 620002934, 620005647, 620006213, 620006214  620006221, 620006222, 620006223, 620008178, 620008179, 620008180  620009528, 621484101, 621484201, 621484501, 621519104, 621519204  621519304, 621536201, 621536301, 621536405, 621536505, 621536605  621536902, 621537002, 621643701, 621645001, 621645101, 621645201  621645402, 621645502, 621660005, 621696901, 621697001, 621697101  621754402, 621808401, 621808501, 621947801, 622082501, 622083101  622093801, 622263401, 622326600, 622326700, 622326800, 622326900  622327000, 622327100, 622462201, 622694000, 622759000, 622759100  622759200, 622759300, 622759400, 622759500, 622759600, 622759700  622759800, 622759900, 622760000, 640463048, 640463049, 640463085  640463086, 620000023, 622011901, 622743900, 622744000 |
| Cilostazol | 610444003, 610444004, 610444026, 610444027, 610444047, 610444048  610444051, 610444056, 610444057, 610444058, 610444059, 610444121  610444122, 610444127, 610444128, 610444129, 610444130, 610444131  610444132, 610444133, 610444140, 610444141, 610444149, 610444150  610453021, 620000049, 620000050, 620002104, 620002105, 620002733  620002734, 620003450, 620003451, 620003610, 620003611, 620005094  620009320, 620009321, 621312902, 621313101, 621313301, 621313502  621313601, 621313702, 621313801, 621314001, 621314101, 621314301  621314601, 621314802, 621314901, 621315002, 621315201, 621315401  621418301, 621418401, 621418501, 621418601, 621418701, 621418802  621523902, 621524002, 621638701, 621721201, 621721301, 621960501  621971201, 621971301, 622088701, 622088801, 622119801, 622226901  622227001, 622256201, 622256301, 622315200, 622315300, 622334101  622334201, 622344001, 622344101, 622346301, 622347901, 622361301  622361401, 622373001, 622373101, 622384101, 622384201, 622501801  622501901 |
| Statins | 610443013, 610443014, 610454084, 610454085, 610462015, 610462016  610470012, 610470013, 610470014, 612180263, 612180264, 612180265  620000013, 620000014, 620000038, 620000039, 620000051, 620000052  620000053, 620000054, 620000070, 620000071, 620000103, 620000104  620000105, 620000106, 620000107, 620000108, 620000109, 620000110  620000111, 620000112, 620000113, 620000114, 620000140, 620000141  620000151, 620000152, 620000153, 620000156, 620000157, 620000158  620000159, 620000160, 620000163, 620000164, 620000169, 620000171  620000174, 620000175, 620000176, 620000177, 620000178, 620000179  620000422, 620000423, 620002049, 620002050, 620002108, 620002109  620002117, 620002118, 620002162, 620002163, 620002164, 620002165  620002166, 620002477, 620002478, 620002736, 620002798, 620002799  620002800, 620002801, 620002802, 620002879, 620002880, 620004038  620004085, 620009322, 620009323, 620009324, 620009325, 621521301  621521401, 621523101, 621523201, 621524102, 621524402, 621525701  621525801, 621528401, 621528501, 621528602, 621528702, 621528801  621528901, 621529001, 621529101, 621531001, 621531101, 621531703  621532401, 621532501, 621532601, 621532902, 621533002, 621533101  621533201, 621533501, 621533601, 621533801, 621533901, 621534003  621534101, 621534204, 621534301, 621623603, 621635202, 621635301  621639001, 621639101, 621639701, 621639801, 621643301, 621643401  621643501, 621643601, 621675101, 621694001, 621752501, 621934801  621934901, 621935001, 621948701, 621948801, 621955001, 621955003  621955101, 621964101, 621964201, 621964301, 621964401, 621964501  621964601, 621981401, 621981403, 622015101, 622015201, 622015301  622052801, 622055601, 622055602, 622062701, 622071601, 622075801  622075901, 622076401, 622076501, 622098401, 622098501, 622099101  622099201, 622102501, 622102502, 622107601, 622107701, 622110401  622110501, 622116801, 622116802, 622116901, 622116902, 622126901  622127001, 622128201, 622128301, 622136401, 622139600, 622143801  622143901, 622152001, 622152101, 622161801, 622161901, 622165601  622165701, 622167601, 622167701, 622169901, 622169902, 622170001  622170002, 622170101, 622170201, 622180601, 622180602, 622180701  622180702, 622186601, 622186701, 622187601, 622187701, 622204801  622204901, 622217101, 622217201, 622239201, 622239301, 622241301  622241401, 622244801, 622244901, 622252001, 622252101, 622268001  622268101, 622268201, 622269101, 622269201, 622270001, 622270101  622271801, 622271901, 622273101, 622273201, 622273301, 622274901  622275001, 622275101, 622276301, 622276401, 622276501, 622280201  622280301, 622280401, 622280501, 622280601, 622280701, 622280801  622282201, 622282301, 622283701, 622283801, 622285001, 622285101  622286201, 622286301, 622286401, 622287601, 622289501, 622289601  622291801, 622291901, 622292001, 622292301, 622292401, 622292501  622293301, 622293401, 622294301, 622294401, 622294501, 622296001  622296101, 622296201, 622297101, 622297201, 622298001, 622298101  622298201, 622299001, 622299101, 622302401, 622302501, 622302801  622302901, 622304601, 622304701, 622304801, 622304901, 622315400  622315500, 622315600, 622321900, 622342801, 622347401, 622359101  622360101, 622362701, 622365801, 622372401, 622387001, 622387601  622392501, 622406901, 622419701, 622419801, 622419901, 622421601  622421701, 622421801, 622426201, 622427701, 622427801, 622431401  622434301, 622434401, 622441101, 622441201, 622457701, 622457801  622457901, 622464901, 622465001, 622465101, 622475000, 622475100  622512001, 622512101, 622512201, 622522101, 622522201, 622522301  622524501, 622524601, 622524701, 622528901, 622529001, 622537301  622537401, 622568601, 622571801, 622571901, 622572801, 622572901  622575201, 622575301, 622575401, 622575501, 622575601, 622575701  622577901, 622578001, 622578101, 622578201, 622578401, 622578501  622578601, 622578701, 622578801, 622581601, 622581701, 622581801  622581901, 622582001, 622582101, 622582501, 622582601, 622582701  622582801, 622584101, 622584201, 622584701, 622584801, 622586001  622586101, 622586201, 622586301, 622588801, 622588901, 622589001  622589101, 622590101, 622590201, 622590301, 622590401, 622591701  622591801, 622591901, 622592001, 622592501, 622592601, 622592701  622592801, 622592901, 622593001, 622593101, 622593201, 622595301  622595401, 622598301, 622598401, 622598501, 622598601, 622599201  622599301, 622599401, 622599501, 622600301, 622600601, 622600701  622600801, 622600901, 622601201, 622601301, 622601401, 622601501  622604001, 622604101, 622604201, 622605001, 622605101, 622605201  622605301, 622605401, 622606601, 622606701, 622615600, 622615700  622615800, 622615900, 622640801, 622640901, 622644801, 622644901  622660001, 622660101, 622665901, 622666001, 622666101, 622676701  622676801, 622691000, 622691800, 622692400, 622692500, 622692600  622692700, 622803401, 622803501, 622803601, 622803701, 622444001  622444101, 622444201, 622444301, 622445401, 622445501, 622445601  622445701, 622447601, 622447701, 622447801, 622447901, 622452401  622452501, 622452601, 622452701, 622453001, 622453101, 622453201  622453301, 622456901, 622457001, 622457101, 622457201, 622461101  622461201, 622461301, 622461401, 622462701, 622462801, 622462901  622463001, 622465301, 622465401, 622465501, 622465601, 622467801  622467901, 622468001, 622468101 |
| Antiplatelet drugs:  Aspirin  Dipyridamole  Ticlopidine  Clopidogrel  Prasugrel | 610431009, 611140017, 611140795, 611140798, 611140849, 611140850  620000484, 620000485, 620000487, 620000488, 620004280, 620008577  620072715, 620072734, 610406235, 610406281, 610422096, 610454004  610454005, 612170034, 612170100, 612170261, 612170310, 612170363  612170397, 620003614, 620003615, 620003616, 620004669, 620006927  620006928, 620008722, 620008786, 620008891, 620008931, 620309504  620309804, 620311004, 620311401, 620311501, 620312301, 620312701  620314401, 620330301, 620330502, 621251701, 622161301, 622161302  622314300, 622726400, 622726500, 622726600, 610407355, 610421088  610443053, 610461073, 613390007, 620000065, 620001952, 620002761  620003275, 620003468, 620003469, 620003599, 620004293, 620004843  620005039, 620005788, 620005812, 620006661, 620007816, 620008315  620008318, 620008330, 620009246, 620009301, 620814301, 620814502  620814505, 620814506, 620814511, 620814512, 620814521, 620814523  620814528, 620814531, 620814533, 620814534, 620814543, 620814601  621374002, 621525202, 621675501, 621676502, 622258001, 622336601  622336701, 622401801, 622401901, 622405801, 622405901, 622406101  622406201, 622407701, 622407801, 622411301, 622413301, 622413401  622413501, 622413601, 622413701, 622414401, 622414501, 622416201  622416301, 622418201, 622418301, 622420101, 622420201, 622420301  622420501, 622420502, 622420601, 622420602, 622422201, 622422301  622424101, 622424201, 622424301, 622425001, 622425101, 622425501  622425601, 622425701, 622427501, 622427601, 622428401, 622428501  622429501, 622429601, 622429701, 622430501, 622430601, 622430701  622431201, 622431301, 622431501, 622431601, 622432801, 622432901  622433001, 622433101, 622433201, 622433301, 622433401, 622434501  622434601, 622435501, 622435601, 622438201, 622438301, 622439501  622439601, 622439801, 622439901, 622452301, 622475400, 622475500  622486701, 622641701, 622641801, 622658901, 622738700 |
| Edaravone | 621974901, 622043501, 622043601, 622044401, 622044701, 622044801  622044901, 622045101, 622048301, 622048601, 622048701, 622051501  622052001, 622052003, 622052501, 622052601, 622056501, 622061901  622061902, 622062001, 622062003, 622063701, 622063801, 622067401  622067501, 622071101, 622071201, 622072401, 622072501, 622074501  622074601, 622074701, 622075201, 622075301, 622077201, 622079501  622079601, 622083001, 622083002, 622083501, 622111201, 622147101  622158501, 622176901, 622220401, 622476800, 622617700, 622692800  622749300, 640451014 |
| Catecholamine | 620002179, 620002180, 620004160, 620005804, 620005858, 620008380  620008381, 620008382, 620244701, 620244702, 620244707, 620244718  620244732, 620244734, 620246104, 620246201, 620246305, 620246306  620246404, 620246501, 620246605, 620246606, 621399006, 621399008  621399013, 621399014, 621644502, 621644602, 622033602, 622043701  622060501, 622060503, 622749400, 622749500, 622749600, 640461007  640461008, 640461009, 640461010, 621371901, 642450005, 642450071  662450001, 622693900 |
| Antihypertensive drug | 610431009, 611140017, 611140795, 611140798, 611140849, 611140850  620000484, 620000485, 620000487, 620000488, 620004280, 620008577  620072715, 620072734, 610406235, 610406281, 610422096, 610454004  610454005, 612170034, 612170100, 612170261, 612170310, 612170363  612170397, 620003614, 620003615, 620003616, 620004669, 620006927  620006928, 620008722, 620008786, 620008891, 620008931, 620309504  620309804, 620311004, 620311401, 620311501, 620312301, 620312701  620314401, 620330301, 620330502, 621251701, 622161301, 622161302  622314300, 622726400, 622726500, 622726600, 610407355, 610421088  610443053, 610461073, 613390007, 620000065, 620001952, 620002761  620003275, 620003468, 620003469, 620003599, 620004293, 620004843  620005039, 620005788, 620005812, 620006661, 620007816, 620008315  620008318, 620008330, 620009246, 620009301, 620814301, 620814502  620814505, 620814506, 620814511, 620814512, 620814521, 620814523  620814528, 620814531, 620814533, 620814534, 620814543, 620814601  621374002, 621525202, 621675501, 621676502, 622258001, 622336601  622336701, 622401801, 622401901, 622405801, 622405901, 622406101  622406201, 622407701, 622407801, 622411301, 622413301, 622413401  622413501, 622413601, 622413701, 622414401, 622414501, 622416201  622416301, 622418201, 622418301, 622420101, 622420201, 622420301  622420501, 622420502, 622420601, 622420602, 622422201, 622422301  622424101, 622424201, 622424301, 622425001, 622425101, 622425501  622425601, 622425701, 622427501, 622427601, 622428401, 622428501  622429501, 622429601, 622429701, 622430501, 622430601, 622430701  622431201, 622431301, 622431501, 622431601, 622432801, 622432901  622433001, 622433101, 622433201, 622433301, 622433401, 622434501  622434601, 622435501, 622435601, 622438201, 622438301, 622439501  622439601, 622439801, 622439901, 622452301, 622475400, 622475500  622486701, 622641701, 622641801, 622658901, 622738700 |
