# Supplementary material for: Fasudil hydrochloride and ozagrel sodium combination therapy for patients with aneurysmal subarachnoid hemorrhage: a cross-sectional study using a nationwide inpatient database
Source: J Pharm Health Care Sci. 2024 Aug 13;10:49. doi: 10.1186/s40780-024-00370-w (PMC11321058; doi:10.1186/s40780-024-00370-w)
Supplement: Supplementary file 3 — Supplementary Material 3 [file 40780_2024_370_MOESM3_ESM.docx]

Additional file 2. Definition of the Japan Coma Scale

| 1-digit code | The patient is awake without any stimuli  (delirium, confusion, senselessness) |
| --- | --- |
| 1 | Almost fully conscious |
| 2 | Unable to recognize time, place, and person |
| 3 | Unable to recall name or date of birth |
| 2-digit code | The patient can be aroused with stimuli  (stupor, lethargy, hypersomnia, somnolence, drowsiness) |
| 10 | By easily by being spoken to (or is responsive with purposeful movements, phrases, or words) |
| 20 | With a loud voice or shaking of shoulders (or is almost always responsive to very simple words like yes or no or to movements) |
| 30 | Only by repeated mechanical stimuli |
| 3-digit code | The patient cannot be aroused with any forceful mechanical stimuli  (deep coma, coma, semicoma) |
| 100 | Responds with movements to avoid the stimulus |
| 200 | Responds with slight movements, including decerebrate and decorticate posture |
| 300 | Does not respond at all except for changes in respiratory rhythm |
