# Supplementary material for: Fasudil hydrochloride and ozagrel sodium combination therapy for patients with aneurysmal subarachnoid hemorrhage: a cross-sectional study using a nationwide inpatient database
Source: J Pharm Health Care Sci. 2024 Aug 13;10:49. doi: 10.1186/s40780-024-00370-w (PMC11321058; doi:10.1186/s40780-024-00370-w)
Supplement: Supplementary file 4 — Supplementary Material 4 [file 40780_2024_370_MOESM4_ESM.docx]

Additional file 3. Definition of ICD-10 codes

| Subarachnoid hemorrhage |  |
| --- | --- |
| I60.0 | Subarachnoid hemorrhage from carotid siphon and bifurcation |
| I60.1 | Subarachnoid hemorrhage from middle cerebral artery |
| I60.2 | Subarachnoid hemorrhage from anterior communicating artery |
| I60.3 | Subarachnoid hemorrhage from posterior communicating artery |
| I60.4 | Subarachnoid hemorrhage from basilar artery |
| I60.5 | Subarachnoid hemorrhage from vertebral artery |
| I60.6 | Subarachnoid hemorrhage from other intracranial arteries |
| I60.7 | Subarachnoid hemorrhage from intracranial artery, unspecified |
| I60.8 | Other subarachnoid hemorrhage |
| I60.9 | Subarachnoid hemorrhage, unspecified |
| Hypertension |  |
| I10 | Essential (primary) hypertension |
| I11 | Hypertensive heart disease |
| I12 | Hypertensive renal disease |
| I13 | Hypertensive heart and renal disease |
| I15 | Secondary hypertension |
| Hyperlipidemia |  |
| E785 | Hyperlipidemia unspecified |
| Diabetes mellitus |  |
| E10 | Type 1 diabetes mellitus |
| E11 | Type 2 diabetes mellitus |
| E12 | Malnutrition-related diabetes mellitus |
| E13 | Other specified diabetes mellitus |
| E14 | Unspecified diabetes mellitus |
| Intracerebral hemorrhage |  |
| I61 | Intracerebral hemorrhage |
| Cerebral infarction |  |
| I63 | Cerebral infarction |
