# Supplementary material for: Fasudil hydrochloride and ozagrel sodium combination therapy for patients with aneurysmal subarachnoid hemorrhage: a cross-sectional study using a nationwide inpatient database
Source: J Pharm Health Care Sci. 2024 Aug 13;10:49. doi: 10.1186/s40780-024-00370-w (PMC11321058; doi:10.1186/s40780-024-00370-w)
Supplement: Supplementary file 5 — Supplementary Material 5 [file 40780_2024_370_MOESM5_ESM.docx]

Additional file 4. Definition of Glasgow Coma Scale

| Japan Coma Scale | Glasgow Coma Scale |
| --- | --- |
| 0 | 15 |
| 1 | 15 |
| 2 | 14 |
| 3 | 13 |
| 10 | 12 |
| 20 | 12 |
| 30 | 9 |
| 100 | 7 |
| 200 | 6 |
| 300 | 3 |
