# Supplementary material for: Fasudil hydrochloride and ozagrel sodium combination therapy for patients with aneurysmal subarachnoid hemorrhage: a cross-sectional study using a nationwide inpatient database
Source: J Pharm Health Care Sci. 2024 Aug 13;10:49. doi: 10.1186/s40780-024-00370-w (PMC11321058; doi:10.1186/s40780-024-00370-w)
Supplement: Supplementary file 6 — Supplementary Material 6 [file 40780_2024_370_MOESM6_ESM.docx]

Additional file 6. Baseline characteristics of patients in the F, FO, and O groups according to age

|  | <75 years | | | | ≥75 years | | | |
| --- | --- | --- | --- | --- | --- | --- | --- | --- |
|  | F group  (n=7,779) | FO group (n=4,854) | O group  (n=335) | p-value | F group  (n=2,705) | FO group (n=1,543) | O group  (n=130) | p-value |
| Age: mean (SD) | 57.4 (11.1) | 57.2 (11.2) | 57.6 (11.3) | 0.419 | 81.4 (4.8) | 81.2 (4.7) | 82.1 (4.9) | 0.110 |
| Sex, n (%) |  |  |  | 0.363 |  |  |  | 0.033 |
| Male | 2793 (35.9) | 1726 (35.6) | 132 (39.4) |  | 364 (13.5) | 212 (13.7) | 28 (21.5) |  |
| Female | 4986 (64.1) | 3128 (64.4) | 203 (60.6) |  | 2341 (86.5) | 1331 (86.3) | 102 (78.5) |  |
| Location of aneurysms, n (%) |  |  |  |  |  |  |  |  |
| ICA | 1986 (25.5) | 1321 (27.2) | 188 (26.3) | 0.111 | 935 (34.6) | 535 (34.7) | 44 (33.8) | 0.982 |
| MCA | 1901 (24.4) | 1083 (22.3) | 56 (16.7) | <0.001 | 555 (20.5) | 329 (21.3) | 27 (20.8) | 0.824 |
| AcomA | 1962 (25.2) | 1302 (26.8) | 83 (24.8) | 0.123 | 566 (20.9) | 344 (22.3) | 30 (23.1) | 0.522 |
| PcomA | 100 (1.3) | 72 (1.5) | 8 (2.4) | 0.186 | 60 (2.2) | 37 (2.4) | 5 (3.8) | 0.474 |
| BA | 266 (3.4) | 184 (3.8) | 24 (7.2) | 0.001 | 131 (4.8) | 66 (4.3) | 6 (4.6) | 0.701 |
| VA | 598 (7.7) | 360 (7.4) | 35 (10.4) | 0.129 | 115 (4.3) | 58 (3.8) | 8 (6.2) | 0.372 |
| Other | 1164 (15.0) | 647 (13.3) | 51 (15.2) | 0.035 | 414 (15.3) | 207 (13.4) | 14 (10.8) | 0.114 |
| Treatment Modality, n (%) |  |  |  | <0.001 |  |  |  | 0.288 |
| Clipping | 4803 (61.7) | 2838 (58.5) | 149 (44.5) |  | 1544 (57.1) | 841 (54.5) | 65 (50.0) |  |
| Coiling | 2858 (36.7) | 1925 (39.7) | 183 (54.6) |  | 1139 (42.1) | 686 (44.5) | 64 (49.2) |  |
| Clipping and coiling | 118 (1.5) | 91 (1.9) | 3 (0.9) |  | 22 (0.8) | 16 (1.0) | 1 (0.8) |  |
| Ambulance use, n (%) | 6689 (86.0) | 4229 (87.1) | 272 (81.2) | 0.005 | 2380 (88.0) | 1364 (88.4) | 111 (85.4) | 0.586 |
| Days from onset of SAH to admission, n (%) |  |  |  | 0.014 |  |  |  | 0.005 |
| ≤3 days | 7534 (96.9) | 4739 (97.6) | 321 (95.8) |  | 2621(96.9) | 1509 (97.8) | 121 (93.1) |  |
| 4-7 days | 245 (3.1) | 115 (2.4) | 14 (4.2) |  | 84 (3.1) | 34 (2.2) | 9 (6.9) |  |
| ICU admission, n (%) | 3618 (46.5) | 2199 (45.3) | 152 (45.4) | 0.404 | 1224 (45.2) | 722 (46.8) | 64 (49.2) | 0.464 |
| Artificial ventilation, n (%) | 4676 (60.1) | 2976 (61.3) | 175 (52.2) | 0.004 | 1683 (62.2) | 952 (61.7) | 68 (52.3) | 0.076 |
| Length of hospital stay (SD) | 44.9 (45.9) | 46.2 (39.2) | 41.5 (38.2) | 0.073 | 57.1 (48.5) | 56.4 (41.4) | 55.2 (40.6) | 0.821 |
| Hospital case volume quartiles, case/4 years, n (%) |  |  |  | <0.001 |  |  |  | <0.001 |
| 1-7 | 315 (4.0) | 186 (3.8) | 25 (7.5) |  | 113 (4.2) | 61 (4.0) | 10 (7.7) |  |
| 8-17 | 940 (12.1) | 741 (15.3) | 84 (25.1) |  | 338 (12.5) | 231 (15.0) | 35 (26.9) |  |
| 18-33 | 2209 (28.4) | 1146 (23.6) | 81 (24.2) |  | 729 (27.0) | 370 (24.0) | 38 (29.2) |  |
| ≥34 | 4315 (55.5) | 2781 (57.3) | 145 (43.3) |  | 1525 (56.4) | 881 (57.1) | 47 (36.2) |  |
| JCS score at admission, n (%) |  |  |  | 0.696 |  |  |  | 0.125 |
| 0 | 1674 (21.5) | 1044 (21.5) | 81 (24.2) |  | 367 (13.6) | 227 (14.7) | 23 (17.7) |  |
| 1-digit code | 2147 (27.6) | 1316 (27.1) | 92 (27.5) |  | 750 (27.7) | 452 (29.3) | 37 (28.5) |  |
| 2-digit code | 1805 (23.2) | 1177 (24.2) | 71 (21.2) |  | 691 (25.5) | 416 (27.0) | 35 (26.9) |  |
| 3-digit code | 2153 (27.7) | 1317 (27.1) | 91 (27.2) |  | 897 (33.2) | 448 (29.0) | 35 (26.9) |  |
| GCS |  |  |  | 0.237 |  |  |  | 0.126 |
| 15 | 2344 (30.1) | 1538 (31.7) | 92 (27.5) |  | 735 (27.2) | 454 (29.4) | 42 (32.3) |  |
| 14 | 360 (4.6) | 248 (5.1) | 15 (4.5) |  | 206 (7.6) | 105 (6.8) | 10 (7.7) |  |
| 13 | 408 (5.2) | 258 (5.3) | 13 (3.9) |  | 176 (6.5) | 120 (7.8) | 8 (6.2) |  |
| 12-7 | 3053 (39.2) | 1854 (38.2) | 145 (43.3) |  | 922 (34.1) | 542 (35.1) | 43 (33.1) |  |
| 6-3 | 1614 (20.7) | 956 (19.7) | 70 (20.9) |  | 666 (24.6) | 322 (20.9) | 27 (20.8) |  |
| mRS score at admission, n (%) |  |  |  | 0.042 |  |  |  | 0.007 |
| 0 | 6178 (79.4) | 3834 (79.0) | 250 (74.6) |  | 1632 (60.3) | 933 (60.5) | 70 (53.8) |  |
| 1 | 735 (9.4) | 479 (9.9) | 47 (14.0) |  | 454 (16.8) | 313 (20.3) | 27 (20.8) |  |
| 2 | 230 (3.0) | 160 (3.3) | 17 (5.1) |  | 201 (7.4) | 107 (6.9) | 15 (11.5) |  |
| 3 | 133 (1.7) | 82 (1.7) | 4 (1.2) |  | 153 (5.7) | 56 (3.6) | 10 (7.7) |  |
| 4 | 188 (2.4) | 107 (2.2) | 2 (0.6) |  | 122 (4.5) | 60 (3.9) | 4 (3.1) |  |
| 5 | 315 (4.0) | 192 (4.0) | 15 (4.5) |  | 143 (5.3) | 74 (4.8) | 4 (3.1) |  |
| Charlson Comorbidity Index, n (%) |  |  |  | 0.068 |  |  |  | <0.001 |
| 0 | 5050 (64.9) | 3246 (66.9) | 225 (67.2) |  | 1590 (58.8) | 1001 (64.9) | 85 (65.4) |  |
| ≥1 | 2729 (35.1) | 1608 (33.1) | 110 (32.8) |  | 1115 (41.2) | 542 (35.1) | 45 (34.6) |  |
| Comorbidities, n (%) |  |  |  |  |  |  |  |  |
| Hypertension | 4359 (56.0) | 2720 (56.0) | 198 (59.1) | 0.536 | 1636 (60.5) | 925 (59.9) | 89 (68.5) | 0.162 |
| Diabetes | 664 (8.5) | 431 (8.9) | 28 (8.4) | 0.785 | 363 (13.4) | 176 (11.4) | 17 (13.1) | 0.165 |
| Hyperlipidemia | 915 (11.8) | 581 (12.0) | 31 (9.3) | 0.328 | 322 (11.9) | 194 (12.6) | 12 (9.2) | 0.490 |
| Cerebral infarction | 478 (6.1) | 371 (7.6) | 26 (7.8) | 0.004 | 139 (5.1) | 100 (6.5) | 12 (9.2) | 0.043 |
| Cerebral hemorrhage | 238 (3.1) | 127 (2.6) | 9 (2.7) | 0.342 | 103 (3.8) | 43 (2.8) | 6 (4.6) | 0.167 |
| Concomitant medication, n (%) |  |  |  |  |  |  |  |  |
| Cilostazol | 3831 (49.2) | 2466 (50.8) | 81 (24.2) | <0.001 | 1210 (44.7) | 743 (48.2) | 27 (20.8) | <0.001 |
| Statins | 2963 (38.1) | 1913 (39.4) | 60 (17.9) | <0.001 | 1013 (37.4) | 645 (41.8) | 25 (19.2) | <0.001 |
| Edaravone | 2255 (29.0) | 1726 (35.6) | 143 (42.7) | <0.001 | 706 (26.1) | 482 (31.2) | 49 (37.7) | <0.001 |
| Catecholamine | 503 (6.5) | 383 (7.9) | 37 (11.0) | <0.001 | 275 (10.2) | 154 (10.0) | 14 (10.8) | 0.951 |
| Antihypertensive drug | 6798 (87.4) | 4256 (87.7) | 249 (74.3) | <0.001 | 2366 (87.5) | 1375 (89.1) | 98 (75.4) | <0.001 |
| Antiplatelet drug | 1853 (23.8) | 1418 (29.2) | 175 (39.1) | <0.001 | 774 (28.6) | 506 (32.8) | 48 (36.9) | 0.004 |

F group: fasudil hydrochloride, FO group: combination of fasudil hydrochloride and ozagrel sodium, O group: ozagrel sodium.

AcomA: anterior communicating artery, BA: basilar artery, GCS: Glasgow Coma Scale, ICA: internal carotid artery, ICU: intensive care unit, JCS: Japan Coma Scale, MCA: middle cerebral artery, mRS: modified Rankin Scale, PcomA: posterior communicating artery, SD: standard deviation, VA: vertebral artery.

Concomitant medications: All medications were administered during hospitalization.

Antihypertensive drug: Antihypertensive agents used for acute treatment after subarachnoid hemorrhage.
