# Supplementary material for: Fasudil hydrochloride and ozagrel sodium combination therapy for patients with aneurysmal subarachnoid hemorrhage: a cross-sectional study using a nationwide inpatient database
Source: J Pharm Health Care Sci. 2024 Aug 13;10:49. doi: 10.1186/s40780-024-00370-w (PMC11321058; doi:10.1186/s40780-024-00370-w)
Supplement: Supplementary file 7 — Supplementary Material 7 [file 40780_2024_370_MOESM7_ESM.docx]

Additional file 7. Baseline characteristics of patients in the F, FO, and O groups according to treatment modality

|  | Clipping | | | | Coiling | | | |
| --- | --- | --- | --- | --- | --- | --- | --- | --- |
|  | F group  (n=6,347) | FO group (n=3,679) | O group  (n=214) | p-value | F group  (n=3,997) | FO group (n=2,611) | O group  (n=247) | p-value |
| Age: mean (SD) | 63.4 (13.9) | 63.1 (13.8) | 65.7 (13.4) | 0.021 | 64.0 (15.2) | 62.8 (15.2) | 63.3 (15.9) | 0.015 |
| Age, n (%) |  |  |  | 0.021 |  |  |  | 0.119 |
| <75 years | 4803 (75.7) | 2838 (77.1) | 149 (69.6) |  | 2858 (71.5) | 1925 (73.7) | 183 (74.1) |  |
| ≥75 years | 1544 (24.3) | 841 (22.9) | 65 (30.4) |  | 1139 (28.5) | 686 (26.3) | 64 (25.9) |  |
| Sex, n (%) |  |  |  | 0.104 |  |  |  | 0.157 |
| Male | 1863 (29.4) | 1034 (28.1) | 73 (34.1) |  | 1249 (31.2) | 870 (33.3) | 85 (34.4) |  |
| Female | 4484 (70.6) | 2645 (71.9) | 141 (65.9) |  | 2748 (68.8) | 1741 (66.7) | 162 (65.6) |  |
| Location of aneurysms, n (%) |  |  |  |  |  |  |  |  |
| ICA | 1638 (25.8) | 1028 (27.9) | 64 (29.9) | 0.037 | 1243 (31.1) | 795 (30.4) | 66 (26.7) | 0.330 |
| MCA | 2185 (34.4) | 1212 (32.9) | 66 (30.8) | 0.207 | 243 (6.1) | 180 (6.9) | 16 (6.5) | 0.417 |
| AcomA | 1528 (24.1) | 901 (24.5) | 53 (24.8) | 0.881 | 968 (24.2) | 715 (27.4) | 60 (24.3) | 0.014 |
| PcomA | 84 (1.3) | 72 (2.0) | 11 (5.1) | <0.001 | 75 (1.9) | 37 (1.4) | 2 (0.8) | 0.204 |
| BA | 38 (0.6) | 22 (0.6) | 2 (0.9) | 0.821 | 351 (8.8) | 224 (8.6) | 28 (11.3) | 0.343 |
| VA | 117 (1.8) | 66 (1.8) | 2 (0.9) | 0.616 | 582 (14.6) | 348 (13.3) | 41 (16.6) | 0.200 |
| Other | 902 (14.2) | 458 (12.4) | 22 (10.3) | 0.017 | 653 (16.3) | 377 (14.4) | 42 (17.0) | 0.097 |
| Ambulance use, n (%) | 5498 (86.6) | 3213(87.3) | 175 (81.8) | 0.055 | 3448 (86.3) | 2286 (87.6) | 205 (83.0) | 0.074 |
| Days from onset of SAH to admission, n (%) |  |  |  | 0.048 |  |  |  | 0.003 |
| ≤3 days | 6171 (97.2) | 3606 (98.0) | 208 (97.2) |  | 3844 (96.2) | 2535 (97.1) | 230 (93.1) |  |
| 4-7 days | 176 (2.8) | 73 (2.0) | 6 (2.8) |  | 153 (3.8) | 76 (2.9) | 17 (6.9) |  |
| ICU admission, n (%) | 3009 (47.4) | 1687 (45.9) | 105 (49.1) | 0.263 | 1756(43.9) | 1178 (45.1) | 109 (44.1) | 0.636 |
| Artificial ventilation, n (%) | 3576 (56.3) | 2168 (58.9) | 96 (44.9) | <0.001 | 2670 (66.8) | 1677 (64.2) | 145 (58.7) | 0.007 |
| Length of hospital stay (SD) | 50.3 (51.2) | 50.9 (40.2) | 53.3 (41.3) | 0.566 | 44.0 (38.8) | 44.8 (39.3) | 38.1 (36.2) | 0.032 |
| Hospital case volume quartiles, case/4 years, n (%) |  |  |  | <0.001 |  |  |  | <0.001 |
| 1-7 | 287 (4.5) | 172 (4.7) | 21 (9.8) |  | 138 (3.5) | 70 (2.7) | 14 (5.7) |  |
| 8-17 | 803 (12.7) | 615 (16.7) | 73 (34.1) |  | 456 (11.4) | 348 (13.3) | 45 (18.2) |  |
| 18-33 | 1842 (29.0) | 911 (24.8) | 42 (19.6) |  | 1062 (26.6) | 577 (22.1) | 76 (30.8) |  |
| ≥34 | 3415 (53.8) | 1981 (53.8) | 78 (36.4) |  | 2341 (58.6) | 1616 (61.9) | 112 (45.3) |  |
| JCS score at admission, n (%) |  |  |  | 0.980 |  |  |  | 0.053 |
| 0 | 1269 (20.0) | 757 (20.6) | 46 (21.5) |  | 750 (18.8) | 494 (18.9) | 58 (23.5) |  |
| 1-digit code | 1739 (27.4) | 1005 (27.3) | 61 (28.5) |  | 1120 (28.0) | 731 (28.0) | 67 (27.1) |  |
| 2-digit code | 1565 (24.7) | 908 (24.7) | 50 (23.4) |  | 893 (22.3) | 653 (25.0) | 53 (21.5) |  |
| 3-digit code | 1774 (28.0) | 1009 (27.4) | 57 (26.6) |  | 1234 (30.9) | 733 (28.1) | 69 (27.9) |  |
| GCS |  |  |  | 0.424 |  |  |  | 0.136 |
| 15 | 2048 (32.3) | 1226 (33.3) | 69 (32.2) |  | 1410 (35.3) | 909 (34.8) | 97 (39.3) |  |
| 14 | 324 (5.1) | 204 (5.5) | 7 (3.3) |  | 258 (6.5) | 161 (6.2) | 12 (4.9) |  |
| 13 | 350 (5.5) | 195 (5.3) | 11 (5.1) |  | 202 (5.1) | 155 (5.9) | 16 (6.5) |  |
| 12-7 | 2334 (36.8) | 1363 (37.0) | 89 (41.6) |  | 1168 (29.2) | 817 (31.3) | 63 (25.5) |  |
| 6-3 | 1291 (20.3) | 691 (18.8) | 38 (17.8) |  | 959 (24.0) | 569 (21.8) | 59 (23.9) |  |
| mRS score at admission, n (%) |  |  |  | 0.002 |  |  |  | 0.006 |
| 0 | 4826 (76.0) | 2720 (73.9) | 150 (70.1) |  | 2884 (72.2) | 1962 (75.1) | 168 (68.0) |  |
| 1 | 660 (10.4) | 478 (13.0) | 35 (16.4) |  | 507 (12.7) | 306 (11.7) | 38 (15.4) |  |
| 2 | 252 (4.0) | 149 (4.1) | 11 (5.1) |  | 175 (4.4) | 110 (4.2) | 20 (8.1) |  |
| 3 | 164 (2.6) | 75 (2.0) | 4 (1.9) |  | 121 (3.0) | 62 (2.4) | 10 (4.0) |  |
| 4 | 174 (2.7) | 86 (2.3) | 3 (1.4) |  | 129 (3.2) | 79 (3.0) | 3 (1.2) |  |
| 5 | 271 (4.3) | 171 (4.6) | 11 (5.1) |  | 181 (4.5) | 92 (3.5) | 8 (3.2) |  |
| Charlson Comorbidity Index, n (%) |  |  |  | <0.001 |  |  |  | 0.368 |
| 0 | 4067 (64.1) | 2507 (68.1) | 154 (72.0) |  | 2490 (62.3) | 1671 (64.0) | 154 (62.3) |  |
| ≥1 | 2280 (35.9) | 1172 (31.9) | 60 (28.0) |  | 1507 (37.7) | 940 (36.0) | 93 (37.7) |  |
| Comorbidities, n (%) |  |  |  |  |  |  |  |  |
| Hypertension | 3671 (57.8) | 2062 (56.0) | 146 (68.2) | 0.001 | 2248 (56.2) | 1521 (58.3) | 140 (56.7) | 0.270 |
| Diabetes | 686 (10.8) | 361 (9.8) | 21 (9.8) | 0.278 | 331 (8.3) | 239 (9.2) | 24 (9.7) | 0.391 |
| Hyperlipidemia | 727 (11.5) | 445 (12.1) | 22 (10.3) | 0.513 | 496 (12.4) | 318 (12.2) | 20 (8.1) | 0.132 |
| Cerebral infarction | 268 (4.2) | 218 (5.9) | 16 (7.5) | <0.001 | 343 (8.6) | 244 (9.3) | 20 (8.1) | 0.516 |
| Cerebral hemorrhage | 225 (3.5) | 100 (2.7) | 6 (2.8) | 0.074 | 114 (2.9) | 68 (2.6) | 9 (3.6) | 0.591 |
| Concomitant medication, n (%) |  |  |  |  |  |  |  |  |
| Cilostazol | 2958 (46.6) | 1856 (50.4) | 42 (19.6) | <0.001 | 2007 (50.2) | 1297 (49.7) | 65 (26.3) | <0.001 |
| Statins | 2414 (38.0) | 1485 (40.4) | 33 (15.4) | <0.001 | 1506 (37.7) | 1031 (39.5) | 51 (20.6) | <0.001 |
| Edaravone | 1906 (30.0) | 1304 (35.4) | 98 (45.8) | <0.001 | 994 (24.9) | 849 (32.5) | 91 (36.8) | <0.001 |
| Catecholamine | 504 (7.9) | 348 (9.5) | 16 (7.5) | 0.027 | 252 (6.3) | 176 (6.7) | 35 (14.2) | <0.001 |
| Antihypertensive drug | 5544 (87.3) | 3206 (87.1) | 155 (72.4) | <0.001 | 3491 (87.3) | 2327 (89.1) | 188 (76.1) | <0.001 |
| Antiplatelet drug | 352 (5.5) | 296 (8.0) | 14 (6.5) | <0.001 | 2204 (55.1) | 1574 (60.3) | 162 (65.6) | <0.001 |

F group: fasudil hydrochloride, FO group: combination of fasudil hydrochloride and ozagrel sodium, O group: ozagrel sodium.

AcomA: anterior communicating artery, BA: basilar artery, GCS: Glasgow Coma Scale, ICA: internal carotid artery, ICU: intensive care unit, JCS: Japan Coma Scale, MCA: middle cerebral artery, mRS: modified Rankin Scale, PcomA: posterior communicating artery, SD: standard deviation, VA: vertebral artery.

Concomitant medications: All medications were administered during hospitalization.

Antihypertensive drug: Antihypertensive agents used for acute treatment after subarachnoid hemorrhage.
