# Supplementary material for: Fasudil hydrochloride and ozagrel sodium combination therapy for patients with aneurysmal subarachnoid hemorrhage: a cross-sectional study using a nationwide inpatient database
Source: J Pharm Health Care Sci. 2024 Aug 13;10:49. doi: 10.1186/s40780-024-00370-w (PMC11321058; doi:10.1186/s40780-024-00370-w)
Supplement: Supplementary file 8 — Supplementary Material 8 [file 40780_2024_370_MOESM8_ESM.docx]

Additional file 8. Baseline characteristics of patients in the F, FO, and O groups according to concomitant medication of cilostazol

|  | Concomitant medication of cilostazol | | | | Non-concomitant medication of cilostazol | | | |
| --- | --- | --- | --- | --- | --- | --- | --- | --- |
|  | F group  (n=5,041) | FO group (n=3,209) | O group  (n=108) | p-value | F group  (n=5,443) | FO group (n=3,188) | O group  (n=357) | p-value |
| Age: mean (SD) | 63.2 (14.3) | 62.5(14.2) | 62.5(15.5) | 0.140 | 64.0 (14.4) | 63.4 (14.4) | 65.0 (14.5) | 0.045 |
| Age, n (%) |  |  |  | 0.642 |  |  |  | 0.035 |
| <75 years | 3831 (76.0) | 2466 (76.8) | 81 (75.0) |  | 3948 (72.5) | 2388 (74.9) | 254 (71.1) |  |
| ≥75 years | 1210 (24.0) | 743 (23.2) | 27 (25.0) |  | 1495 (27.5) | 800 (25.1) | 103 (28.9) |  |
| Sex, n (%) |  |  |  | 0.175 |  |  |  | 0.359 |
| Male | 1518 (30.1) | 951 (29.6) | 41 (38.0) |  | 1639 (30.1) | 987 (31.0) | 119 (33.3) |  |
| Female | 3523 (69.9) | 2258 (70.4) | 67 (62.0) |  | 3804 (69.9) | 2201 (69.0) | 238 (66.7) |  |
| Location of aneurysms, n (%) |  |  |  |  |  |  |  |  |
| ICA | 1411 (28.0) | 973 (30.3) | 37 (34.3) | 0.036 | 1510 (27.7) | 883 (27.7) | 95 (26.6) | 0.898 |
| MCA | 1152 (22.9) | 671 (20.9) | 14 (13.0) | 0.009 | 1304 (24.0) | 741 (23.2) | 69 (19.3) | 0.122 |
| AcomA | 1240 (24.6) | 836 (26.1) | 21 (19.4) | 0.131 | 1288 (23.7) | 810 (25.4) | 92 (25.8) | 0.156 |
| PcomA | 89 (1.8) | 60 (1.9) | 4 (3.7) | 0.324 | 71 (1.3) | 49 (1.5) | 9 (2.5) | 0.145 |
| BA | 192 (3.8) | 126 (3.9) | 8 (7.4) | 0.160 | 205 (3.8) | 124 (3.9) | 22 (6.2) | 0.077 |
| VA | 323 (6.4) | 188 (5.9) | 11 (10.2) | 0.142 | 390 (7.2) | 230 (7.2) | 32 (9.0) | 0.444 |
| Other | 745 (14.8) | 420 (13.1) | 16 (14.8) | 0.097 | 833 (15.3) | 434 (13.6) | 49 (13.7) | 0.089 |
| Treatment Modality, n (%) |  |  |  | 0.001 |  |  |  | <0.001 |
| Clipping | 2958 (58.7) | 1856 (57.8) | 42 (38.9) |  | 3389 (62.3) | 1823 (57.2) | 172 (48.2) |  |
| Coiling | 2007 (39.8) | 1297 (40.4) | 65 (60.2) |  | 1990 (36.6) | 1314 (41.2) | 182 (51.0) |  |
| Clipping and coiling | 76 (1.5) | 56 (1.7) | 1 (0.9) |  | 64 (1.2) | 51 (1.6) | 3 (0.8) |  |
| Ambulance use, n (%) | 4406 (87.4) | 2835 (88.3) | 94 (87.0) | 0.433 | 4663 (85.7) | 2758 (86.5) | 289 (81.0) | 0.016 |
| Days from onset of SAH to admission, n (%) |  |  |  | 0.039 |  |  |  | 0.011 |
| ≤3 days | 4895 (97.1) | 3144 (98.0) | 104 (96.3) |  | 5260 (96.6) | 3104 (97.4) | 338 (94.7) |  |
| 4-7 days | 146 (2.9) | 65 (2.0) | 4 (3.7) |  | 183 (3.4) | 84 (2.6) | 19 (5.3) |  |
| ICU admission, n (%) | 2359 (46.8) | 1421 (44.3) | 57 (52.8) | 0.029 | 2483 (45.6) | 1500 (47.1) | 159 (44.5) | 0.364 |
| Artificial ventilation, n (%) | 2985 (59.2) | 2011 (62.7) | 67 (62.0) | 0.007 | 3374 (62.0) | 1917 (60.1) | 176 (49.3) | <0.001 |
| Length of hospital stay (SD) | 46.4 (41.3) | 47.8 (37.3) | 47.8 (40.7) | 0.280 | 49.6 (51.4) | 49.5 (42.4) | 44.6 (38.9) | 0.158 |
| Hospital case volume quartiles, case/4 years, n (%) |  |  |  | <0.001 |  |  |  | <0.001 |
| 1-7 | 194 (3.8) | 95 (3.0) | 13 (12.0) |  | 234 (4.3) | 152 (4.8) | 22 (6.2) |  |
| 8-17 | 485 (9.6) | 444 (13.8) | 21 (19.4) |  | 793 (14.6) | 528 (16.6) | 98 (27.5) |  |
| 18-33 | 1412 (28.0) | 663 (20.7) | 20 (18.5) |  | 1526 (28.0) | 853 (26.8) | 99 (27.7) |  |
| ≥34 | 2950 (58.5) | 2007 (62.5) | 54 (50.0) |  | 2890 (53.1) | 1655 (51.9) | 138 (38.7) |  |
| JCS score at admission, n (%) |  |  |  | 0.492 |  |  |  | 0.332 |
| 0 | 970 (19.2) | 621 (19.4) | 26 (24.1) |  | 1071 (19.7) | 650 (20.4) | 78 (21.8) |  |
| 1-digit code | 1407 (27.9) | 853 (26.6) | 29 (26.9) |  | 1490 (27.4) | 915 (28.7) | 100 (28.0) |  |
| 2-digit code | 1250 (24.8) | 851 (26.5) | 26 (24.1) |  | 1246 (22.9) | 742 (23.3) | 80 (22.4) |  |
| 3-digit code | 1414 (28.0) | 884 (27.5) | 27 (25.0) |  | 1636 (30.1) | 881 (27.6) | 99 (27.7) |  |
| GCS |  |  |  | 0.172 |  |  |  | 0.230 |
| 15 | 1812 (35.9) | 1125 (35.1) | 46 (42.6) |  | 1976 (36.3) | 1183 (37.1) | 141 (39.5) |  |
| 14 | 311 (6.2) | 183 (5.7) | 3 (2.8) |  | 303 (5.6) | 180 (5.6) | 20 (5.6) |  |
| 13 | 254 (5.0) | 166 (5.2) | 6 (5.6) |  | 282 (5.2) | 202 (6.3) | 17 (4.8) |  |
| 12-7 | 1595 (31.6) | 1102 (34.3) | 31 (28.7) |  | 1671 (30.7) | 978 (30.7) | 104 (29.1) |  |
| 6-3 | 1069 (21.2) | 633 (19.7) | 22 (20.4) |  | 1211 (22.2) | 645 (20.2) | 75 (21.0) |  |
| mRS score at admission, n (%) |  |  |  | 0.248 |  |  |  | 0.014 |
| 0 | 3855 (76.5) | 2438 (76.0) | 81 (75.0) |  | 3955 (72.7) | 2329 (73.1) | 239 (66.9) |  |
| 1 | 530 (10.5) | 378 (11.8) | 11 (10.2) |  | 659 (12.1) | 414 (13.0) | 63 (17.6) |  |
| 2 | 191 (3.8) | 135 (4.2) | 8 (7.4) |  | 240 (4.4) | 132 (4.1) | 24 (6.7) |  |
| 3 | 134 (2.7) | 66 (2.1) | 3 (2.8) |  | 152 (2.8) | 72 (2.3) | 11 (3.1) |  |
| 4 | 146 (2.9) | 76 (2.4) | 2 (1.9) |  | 164 (3.0) | 91 (2.9) | 4 (1.1) |  |
| 5 | 185 (3.7) | 116 (3.6) | 3 (2.8) |  | 273 (5.0) | 150 (4.7) | 16 (4.5) |  |
| Charlson Comorbidity Index, n (%) |  |  |  |  |  |  |  |  |
| 0 |  |  |  |  |  |  |  |  |
| ≥1 | 1856 (36.8) | 1028 (32.0) | 44 (40.7) | <0.001 | 1988 (36.5) | 1122 (35.2) | 111 (31.1) | 0.075 |
| Comorbidities, n (%) |  |  |  |  |  |  |  |  |
| Hypertension | 2872 (57.0) | 1857 (57.9) | 69 (63.9) | 0.283 | 3123 (57.4) | 1788 (56.1) | 218 (61.1) | 0.150 |
| Diabetes | 515 (10.2) | 276 (8.6) | 17 (15.7) | 0.005 | 512 (9.4) | 331 (10.4) | 28 (7.8) | 0.162 |
| Hyperlipidemia | 726 (14.4) | 457 (14.2) | 13 (12.0) | 0.778 | 511 (9.4) | 318 (10.0) | 30 (8.4) | 0.503 |
| Cerebral infarction | 368 (7.3) | 301 (9.4) | 14 (13.0) | 0.001 | 249 (4.6) | 170 (5.3) | 24 (6.7) | 0.081 |
| Cerebral hemorrhage | 146 (2.9) | 57 (1.8) | 2 (1.9) | 0.005 | 195 (3.6) | 113 (3.5) | 13 (3.6) | 0.993 |
| Concomitant medication, n (%) |  |  |  |  |  |  |  |  |
| Statins | 2672 (53.0) | 1690 (52.7) | 25 (23.1) | <0.001 | 1304 (24.0) | 868 (27.2) | 60 (16.8) | <0.001 |
| Edaravone | 1620 (32.1) | 1210 (37.7) | 52 (48.1) | <0.001 | 1341 (24.6) | 998 (31.3) | 140 (39.2) | <0.001 |
| Catecholamine | 321 (6.4) | 272 (8.5) | 13 (12.0) | <0.001 | 457 (8.4) | 265 (8.3) | 38 (10.6) | 0.314 |
| Antihypertensive drug | 4444 (88.2) | 2866 (89.3) | 80 (74.1) | <0.001 | 4720 (86.7) | 2765 (86.7) | 267 (74.8) | <0.001 |
| Antiplatelet drug | 1202 (23.8) | 808 (25.2) | 50 (46.3) | <0.001 | 1425 (26.2) | 1116 (35.0) | 129 (36.1) | <0.001 |

F group: fasudil hydrochloride, FO group: combination of fasudil hydrochloride and ozagrel sodium, O group: ozagrel sodium.

AcomA: anterior communicating artery, BA: basilar artery, GCS: Glasgow Coma Scale, ICA: internal carotid artery, ICU: intensive care unit, JCS: Japan Coma Scale, MCA: middle cerebral artery, mRS: modified Rankin Scale, PcomA: posterior communicating artery, SD: standard deviation, VA: vertebral artery.

Concomitant medications: All medications were administered during hospitalization.

Antihypertensive drug: Antihypertensive agents used for acute treatment after subarachnoid hemorrhage.
